# Building Block Analysis of ATIII Affinity Fractions of Heparins: Application to the ATIII Binding Capacity of Non-conventional 3-*O*-Sulfated Sequences

**DOI:** 10.3389/fmed.2022.841738

**Published:** 2022-04-19

**Authors:** Pierre Mourier

**Affiliations:** Sanofi Chimie, Aramon, France

**Keywords:** heparinase digestion, 3-*O*-sulfated disaccharides, sulfanilic tagging, heparin, ATIII affinity

## Abstract

In heparin, some 3-*O*-sulfated sequences do not meet the structural requirements of the ATIII binding pentasaccharide. These “non-conventional” sequences are the object of this study. In a previous paper (Mourier P. Heparinase digestion of 3-*O*-sulfated sequences: selective heparinase II digestion for separation and identification of binding sequences present in ATIII affinity fractions of bovine intestine heparins), we demonstrated that unsaturated 3-*O*-sulfated disaccharides detected in exhaustive heparin digests were specifically cleaved by heparinase I. Consequently, building blocks analyses of heparins using heparinases I+II+III digestion could be compared with experiments where only heparinase II is used. In these latter conditions of depolymerization, the 3-*O*-sulfated sequences digested into unsaturated 3-*O*-sulfated disaccharides with heparinases I+II+III, were heparinase II-resistant on their non-reducing side, resulting in longer new building blocks. These properties were used to study the structural neighborhood of these 3-*O*-sulfated moieties, which have still-undefined biological functions. In this part, heparinases I+II+III and heparinase II digestions of porcine mucosa, bovine mucosa and bovine lung heparins were compared in six fractions of increasing affinity for ATIII. Tagging of building blocks by reductive amination with sulfanilic acid was used. The distribution of 3-*O*-sulfated building blocks in the ATIII affinity fractions was used to examine the ATIII binding of these sequences.

## Introduction

Heparin is a linear polymeric chain with repeated sulfated disaccharide units, which has been used since the early 1930s as an antithrombotic anticoagulant in cardiovascular medicine. Heparin used in the United States and European countries is exclusively extracted from porcine intestinal mucosa and is also the starting material for the synthesis of low molecular weight heparins (LMWH). There is a serious risk of global shortage of porcine heparin due to rising demand and more recently to African swine fever, resulting in a continuous increase of its price for more than 10 years. This risk is a serious concern for drug agencies and the United States Pharmacopeia, and since August 2015 (6th Workshop on the characterization of heparin products, São Paulo), there has been a focus for research into heparins prepared from other species and tissues. However, the structural and biological equivalence of these heparins has not been confirmed, and heparins from different sources, particularly in the case of bovine and intestinal mucosa, are regarded as different products (1). The comparison of bovine and porcine mucosa heparins (BMH and PMH, respectively) is particularly interesting; PMH have equivalent anticoagulant activities, irrespective of the method used (2), but BMH have lower specific Anti-Xa (AXa) and Anti-IIa (AIIa) activities than PMH, with a higher AIIa/AXa ratio (1.2 vs. 1) and substantial differences in activity values depending on the assay method. Thus, the anticoagulant activities of BMH and PMH are different in both extent and mechanism.

In the first part of this study (3), the presence of unsaturated 3-*O*-sulfated (3S) disaccharides in heparin after digestion by heparinases I+II+III was shown to result from the specific cleavage of the non-reducing side of non-conventional 3S sequences by heparinase I, possible either with 2-*O*-sulfated uronic acids [ΔIs (ΔHexUA(2S)-GlcN(NS,3S,6S)], ΔIIIs [ΔHexUA(2S)-GlcN(NS,3S)] or with conjugated 3S disaccharides [ΔIIs (ΔHexUA-GlcN(NS,3S,6S)] (structural symbols are listed in Table 1). Two methods for building block analysis of heparin were then realized: exhaustive heparinases I+II+III digestion, and a more specific mode with only heparinase II. In the latter case, in the absence of heparinase I the unsaturated 3S disaccharides could not be generated, resulting in new building blocks (mainly tetra- and hexasaccharides). For the first two disaccharides, ΔIs and ΔIIIs, incompatibility with the structural requirements of the antithrombin III (ATIII) binding sequence suggested that they did not participate in anticoagulant activity and could be classified as non-ATIII-binding units (4). However, recent studies based on biosynthetic sequences (5, 6), showed that the medium glucuronic acid in the pentasaccharide is not mandatory to bind ATIII and could be replaced by a 2-*O*-sulfated iduronic acid in synthetic octasaccharides, which still have anticoagulant activity. ΔIs and ΔIIIs could then be digested from ATIII binding sequences where their uronic acid is iduronic 2-*O*-sulfate [-IdoA(2S)-GlcN(NS,3S,6S) and -IdoA(2S)-GlcN(NS,3S)].

**TABLE 1:** Nomenclature and structural symbols.

Nomenclature	
HexUA, Uronic acid	IdoA, L-iduronic acid
GlcA, D-glucuronic acid	ΔHexUA, 4,5-unsaturated uronic acid
GlcN, D-glucosamine	NAc, *N*-acetyl
NS, *N*-sulfate	2S, 2-*O*-sulfate
GalA, D-galacturonic acid	6S, 6-*O*-sulfate
PMH, porcine mucosa heparin	3S, 3-*O*-sulfate
BMH, bovine mucosa heparin	w/w, weight/weight
BLH, bovine lung heparin	

**Structural symbols**	

ΔIVa, ΔHexUA-GlcNAc	ΔIVs, ΔHexUA-GlcNS
ΔIIa, ΔHexUA-GlcNAc(6S)	ΔIIIa, ΔHexUA(2S)-GlcNAc
ΔIIs, ΔHexUA-GlcN(NS,6S)	ΔIIIs, ΔHexUA(2S)-GlcNS
ΔIa, ΔHexUA(2S)-GlcNAc(6S)	ΔIs, ΔHexUA(2S)-GlcN(NS,6S)
ΔIIs, ΔHexUA-GlcN(N*S*,3S,6S)	ΔIIIs, ΔHexUA(2S)-GlcN(NS,3S)
ΔIs, ΔHexUA(2S)-GlcN(NS,3S,6S)	IVs_gal_, GalA-GlcNS
IIs_gal_, GalA-GlcN(NS,6S)	IIIs_id_, IdoA(2S)-GlcNS
IIs_glu_, GlcA-GlcN (NS,6S)	Is_id_, IdoA(2S)-GlcN(NS,6S)
IVs_glu_, GlcA-GlcNS	Is_id_, IdoA(2S)-GlcN(NS,3S,6S)
Is_glu_, GlcA(2S)-GlcN(NS,3S,6S)	IIIs_id_, IdoA(2S)-GlcN(NS,3S)
IIs_glu_, GlcA-GlcN(NS,3S,6S)	IVs_glu_, GlcA-GlcN(NS,3S)
Glyserox, oxidized glycoserine (ΔGlcA-Gal-Gal-Xyl-COOH)	
ΔU(x,y,z), Δ-unsaturated oligosaccharide, x saccharides units, y sulfates,	
z *N*-acetyl	
U(x,y,z) = saturated oligosaccharide, x saccharides units, y sulfates,	
z *N*-acetyl	
ΔU(x,y,z)^sulf^, ΔU(x,y,z) with sulfanilic acid reductive amination	
G(x,y,z), oligosaccharide with a glucosamine at its non-reducing end,	
x saccharides units, y sulfates, z *N*-acetyl	
G(x,y,z)^sulf^, G(x,y,z) with sulfanilic acid reductive amination	
Mw 595^sulf^, Oligosaccharide at Mw595Da with sulfanilic reductive amination	
(595 + 157Da)	
The iduronic (id) or glucuronic (glu) structure of uronic acids is indicated for	
oligosaccharides, e.g., ΔIs-III_id_	
Underlined disaccharides have a 3-*O*-sulfated glucosamine, e.g.,	
IIs_*glu*_ (GlcA-GlcNS,3S,6S)	

Consequently, there is obvious uncertainty surrounding the contribution of sequences including IdoA(2S)-GlcN(NS,3S,6OH/S) to the anticoagulant activity of heparins, which is particularly acute for bovine heparins. To investigate this question, building block analyses of heparin fractions with various affinities to ATIII, obtained from ATIII affinity chromatography, were performed using three types of heparins, PMH, BMH, and bovine lung heparin (BLH), by exhaustive heparinase digestion with the heparinases I+II+III mixture and specific digestion with heparinase II only. The building blocks were then quantified after sulfanilic tagging (7).

## Materials and Methods

### Materials

Porcine mucosa heparin was obtained from Scientific Protein Laboratories (Madison, Wisconsin). Bovine intestinal heparin was obtained from Opocrin (LDO Spa, Milano, Italy) and the BLH was a special batch purified by Bioiberica (Barcelona, Spain). All enzyme lyases from *Flavobacterium heparinum* [Heparinase I (EC 4.2.2.7), Heparinase II (no EC number), Heparinase III (EC 4.2.2.8)] were obtained from Grampian Enzymes (Aberdeen). All other reagents and chemicals were of the highest quality available. Water was purified using a Millipore Milli-Q purification system.

### Heparin Fractionation

#### Antithrombin Affinity Chromatography

An ATIII–Sepharose column (30 × 7 cm) prepared by coupling 2 g of human ATIII to CNBr-activated Sepharose 4B (Sigma) as described by Höök and coworkers (8) was used. A step gradient of NaCl concentration was applied. Low-affinity fractions were eluted using a 0.25 M NaCl solution buffered at pH 7.4 with 1 mM Tris–HCl at 11 ml/min; high-affinity fractions were eluted by a five steps gradient of NaCl, typically (0.74, 1.23, 1.71, 2.2, and 3.5 M NaCl and 1 mM Tris–HCl, pH 7.4). The NaCl gradient was monitored by conductivity measurements, and the heparin fractions were detected in the UV (Ultraviolet) at 219 nm. This wavelength, with a limited influence of the NaCl present in the mobile phase, is sensitive to *N*-acetyl functions present all over the heparin chain. The chromatograms of the separation were obtained after subtraction of the signal obtained on a blank run. Injected quantities could vary between 250 mg and 500 mg of heparin depending on the capacity of the column and the type of heparin injected.

#### Desalting

Multiple desalting steps were mandatory to eliminate NaCl, especially for fractions of highest affinity. First, high-affinity fractions (1 L) were diluted 1/5 in water and passed through a 20 × 1.6 cm column filled with Q-Sepharose Fast Flow (Sigma Aldrich, Saint-Quentin-Fallavier, France). The column was then washed with water to eliminate free sodium chloride. The heparin was then flash eluted by NaClO_4_ 2.5 N. UV detection at 215 nm was used to monitor the elution. In a second step, the heparin solution was desalted on a 100 × 7 cm column filled with Sephadex G10 monitored with UV detection at 215 nm and conductimetry.

### Enzymatic Digestion

All heparinases were at 0.5 IU/mL in a pH 7.0 potassium phosphate buffer [10 mM KH_2_PO_4_ and 0.2 mg/mL of bovine serum albumin (BSA)]. Depolymerizations with heparinase II and heparinases I+II+III were performed at room temperature for 48 h in a total volume of 170 μL containing 20 μL of a 20 mg/mL solution of heparin in water, 20 μL of a mixture of the heparinases at 0.5 IU/mL and 130 μL of 100 mM sodium acetate buffer (pH 7.0) containing 2 mM Ca(OAc)_2_ and 0.5 mg/mL BSA.

### Reductive Amination With Sulfanilic Acid

Heparin building blocks generated by the digestion of heparins with heparinase were tagged by sulfanilic acid, as previously described (7). Oligosaccharides obtained after digestion were diluted to 200 μL with 4% acetic acid (v/v in water). They were introduced into an HPLC vial (1.7 mL) containing 4–6 mg of sulfanilic acid and 6–10 mg of picoline borane. The reaction was complete after 8 h at 37°C. The remaining reagents were removed on Sephadex G10 (column 30 × 2.6 cm) circulated with H_2_O/EtOH, 90/10, v/v.

### Chromatographic Analysis of Digests

#### Strong Anion Exchange Chromatography on AS11 Columns

Two chromatographic AS11 columns (25 × 0.21 cm) (Thermo Scientific Dionex, Montigny-le-Bretonneux, France) were connected in series. The column temperature was set at 40°C. Mobile phase A was 2.5 mM NaH_2_PO_4_ at pH 3.2, and mobile phase B was an aqueous solution of 2.5 mM NaH_2_PO_4_ with 1 M NaClO_4_ adjusted to pH 3.0. A linear gradient (t0 min B% 0; t 80 min B% 60) was applied for elution at a flow rate of 0.22 mL/min. Diode array detection was used. Double UV detection was performed at 265 nm and 232 nm. An NRE building block-specific signal was obtained by the reconstruction of 265 nm - 2.21 × 232 nm (7).

## Results and Discussion

### Analysis of Heparin Fractions With Varied Affinity for Antithrombin III: Exhaustive Versus Specific Heparinase II Digests

The first part of this study compared building blocks from heparinases I+II+III and heparinase II digests of BMH and its ATIII affinity fractions. The digests obtained were complex, with structural diversity due to high 6-*O*-desulfatation and the presence of multiple 3-*O*-sulfated sequences, including non-conventional sequences not meeting the structural requirements of the ATIII binding pentasaccharide (9). These sequences revealed interesting information on the structural environment of the 3S disaccharides. Classic ATIII binding sites containing the key trisaccharide GlcN(NS/NAc,6OH/6S)-GlcA-GlcN(NS,3S,6OH/6S) were digested by heparinases into an unsaturated 3S tetrasaccharides, however, even within purely endogenous fragments, not all tetrasaccharide building blocks generated by heparinases I+II+III BMH digests have been identified. While the question of the ATIII binding of these heparin sequences, including the 3S disaccharides, is still pending, it seems likely that they should be at least 3S. It is obviously not a major issue for PMH, since these sequences comprise less than 10% of the 3S moieties (7). In bovine heparins, their content is much higher and can rise to over 50% in BMH and 30–40% in BLH.

In our laboratory, the development of ATIII affinity chromatography was initiated almost 20 years ago, as a tool for the structural analysis of LMWH and particularly enoxaparin (10–12). It was also used to fractionate heparins to study the structure of fractions with the highest affinity for ATIII. Their measured AXa and AIIa activities could be more than four times that of the starting heparin, outperforming the classic ATIII binding pentasaccharide. The isolation at gram scale of high-affinity fractions for PMH, BMH and BLH (Table 2) was performed to obtain significant amounts of these fractions present at about 1.5% of the starting heparin. It was therefore decided to use these fractions to clarify the contribution of 3S disaccharides to affinity for ATIII, with a focus on bovine heparins. Indeed, the ATIII high-affinity fractions, concentrating the heparin 3S derivatives, were the best fractions to use with selective heparinase II depolymerization to decipher their structural environment.

**TABLE 2:** Mean molecular weights (Mw) and activities (on dry basis) of the starting heparins.

	Mw (Da)	AXa (IU/mg)	AIIa (IU/mg)
**PMH**	–	211	212
**BMH**	17,100	133	136
**BLH**	13,600	144	133

*BLH, bovine lung heparin; BMH, bovine mucosa heparin; PMH, porcine mucosa heparin.*

#### Affinity Chromatography on Immobilized Antithrombin III

**FIGURE 1:**
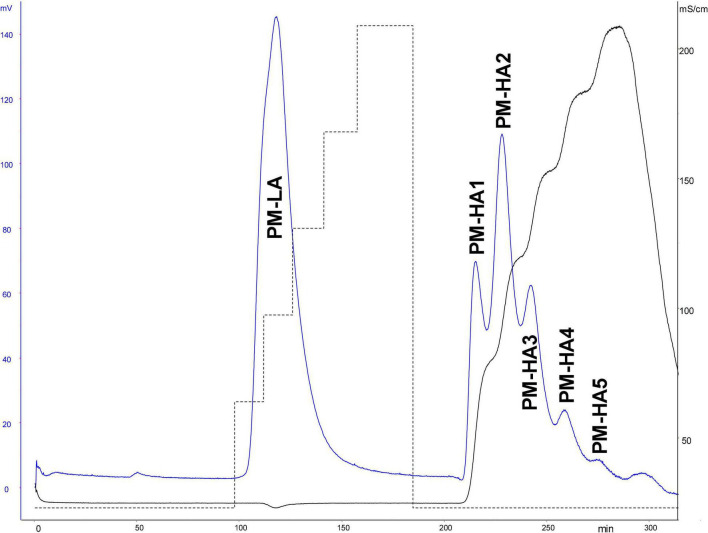
ATIII affinity chromatography of porcine mucosa heparin (PMH). Detection: UV 219 nm and conductimetry.

Antithrombin III affinity chromatography was used to fractionate heparins into an ATIII low-affinity fraction (LA) and five fractions of increasing affinity for ATIII (HA1 to HA5) (Figure 1) as previously described (3), using 475 mg injections of PMH. The applied five-step concentration gradient (0.97, 1.55, 2.14, 2.79, and 3.5 M NaCl) was different to that previously used for BMH (3) because it was implemented on a recently bonded affinity column. The gradient was adapted to keep the HA fraction structurally constant. The capacity and retention of ATIII affinity columns continuously decrease during their lifecycle, and care must be taken to avoid column overloading, otherwise the content of the affinity fraction would be affected. The steps were adapted to the evolution of the column by an analytical control (building block analysis) of the affinity fractions. The object of the step gradient is to split the affinity fractions of heparin in a repeatable way. However, it is difficult to ensure a complete equivalence of the ATIII affinity of respectively collected fractions with the three heparins at different chromatographic states of the affinity column. A comparison of the percentages of the affinity fractions (measured w/w on 1.5 g of heparin through the weight of desalted fractions) under equivalent experimental conditions obtained values of 42.7, 34.7, and 29.9% for PMH, BMH, and BLH, respectively. The properties of the three heparins used for this study are shown in Table 2, and those from the collected fractions in Table 3. The AXa and AIIa activities of the fractions were measured for PMH and showed a continuous increase of activity with increasing ATIII affinity associated with a decreasing mean molecular weight.

**TABLE 3:** Mean molecular weights (Mw) and activities (on dry basis) of the fractions collected on ATIII affinity chromatography of the studied heparins with their quantities and their concentration in heparin.

	Qty (g)	% (w/w)	Mw (Da)	AXa IU/mg	AIIa IU/mg		Qty (g)	% (w/w)	Mw (Da)		Qty (g)	% (w/w)	Mw (Da)
				
PMH						BMH				BLH			
**LA**	7.6	55.8	14,600	56	52	**LA**	14.2	63.9	15,100	**LA**	9.19	63.9	12,000
**HA1**	1.36	10	18,250	283	279	**HA1**	1.63	7.3	19,800	**HA1**	0.99	6.9	18,400
**HA2**	2.54	18.7	17,200	397	391	**HA2**	2.56	11.5	19,600	**HA2**	2.0	13.9	17,600
**HA3**	1.36	10	16,700	528	564	**HA3**	2.14	9.7	18,300	**HA3**	1.40	9.7	15,000
**HA4**	0.55	4	16,600	570	752	**HA4**	1.17	5.3	20,300	**HA4**	0.59	4.1	16,600
**HA5**	0.21	1.6	15,700	608	883	**HA5**	0.50	2.3	18,500	**HA5**	0.20	1.4	15,700

*Qty, quantity; BLH, bovine lung heparin; BMH, bovine mucosa heparin; PMH, porcine mucosa heparin.*

#### Building Block Analysis of Affinity Fractions With Exhaustive Heparinase I+II+III Digestion and Tagging by Sulfanilic Acid

**FIGURE 2:**
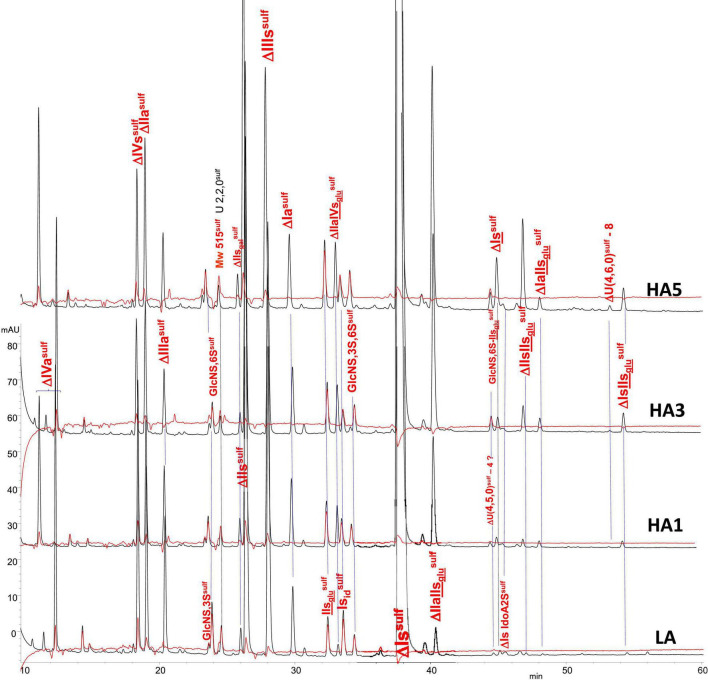
Chromatogram on AS11 of exhaustive heparinase digests from porcine mucosa heparin (PMH) affinity fractions with sulfanilic tagging. Detection: (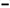) 265 nm; (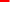) 265 nm – 2.21 × 232 nm (UV selective saturated NRE signal).

**FIGURE 3:**
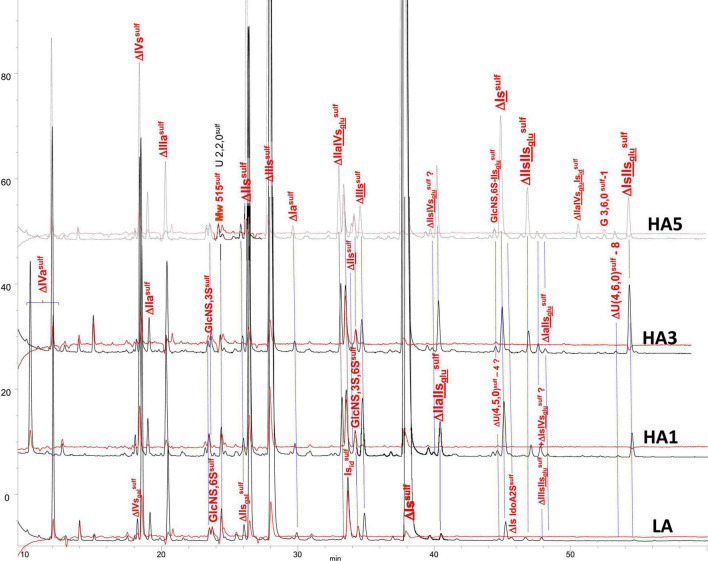
Chromatogram on AS11 of exhaustive heparinase digests from bovine mucosa heparin (BMH) affinity fractions with sulfanilic tagging. Detection: (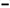) 265 nm; (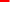) 265 nm – 2.21 × 232 nm (UV selective saturated NRE signal).

**FIGURE 4:**
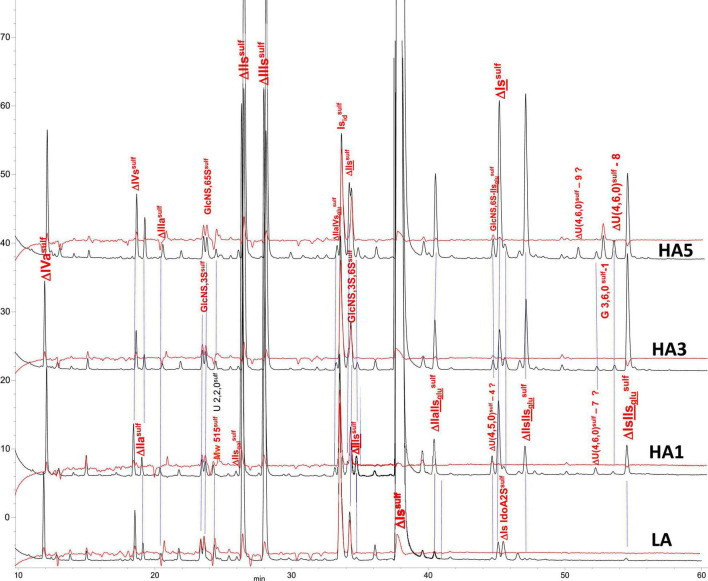
Chromatogram on AS11 of exhaustive heparinase digests from bovine lung heparin (BLH) affinity fractions with sulfanilic tagging. Detection: (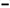) 265 nm; (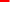) 265 nm – 2.21 × 232 nm (UV selective saturated NRE signal).

Heparin fractions were characterized by analysis of the building blocks generated by exhaustive heparinase digestion. Reductive amination with sulfanilic acid was performed before chromatographic analysis (7), and sulfanilic tagged derivatives were indicated with the superscript ^sulf^. This new assay method is more selective than the classical method and includes non-reducing end (NRE) building blocks that are not accessible with the classical method (13) since they do not contain any Δ^4–5^ unsaturated acids. The major derivatives identified were the four glucosamines GlcNS^sulf^, GlcN(NS,6S)^sulf^, GlcN(NS,3S)^sulf^, GlcN(NS,3S,6S)^sulf^ and the three NRE disaccharides Is_id_^sulf^ [IdoA(2S)-GlcN(NS,6S)^sulf^], IIs_glu_^sulf^ ([GlcA-GlcN(NS,3S,6S)^sulf^] and U(2,2,0)^sulf^. Detection was performed at 265 nm and a signal specific of NRE building blocks (265 nm – 2.21 × 232 nm) was implemented (7). Tables 4–6 show the analytical results obtained respectively with PMH, BMH and BLH. Figures 2–4 shows the corresponding chromatograms obtained respectively for the three heparin sources. Each figure shows chromatograms of separation of building blocks on AS11 columns for a single heparin source and compared results when the affinity for ATIII of the analyzed heparin fraction is modified, that is for LA, HA1, HA3, and HA5 fraction. Another view of the same chromatograms is given in the Supplementary Material, where the influence of the animal origin of the three heparins (PMH, BMH, and BML) is shown for each type of affinity fraction (LA, HA1, HA3, and HA5) (Supplementary Figures 1–4). The quantified building blocks were those described previously (7), including the eight classical disaccharides (Table 1), the two galacturonic acid disaccharides (ΔIVs_gal_ and ΔIIs_gal_), the 3S disaccharides (ΔIs, ΔIIs, and ΔIIIs) and the five main 3S tetrasaccharides (ΔIIa-IVs_glu_, ΔIIa-IIs_glu_, ΔIIs-IIs_glu_, ΔIa-IIs_glu_, and ΔIs-IIs_glu_). The 3S tetrasaccharides evidenced in ion-pair liquid chromatography–mass spectrometry (LC/MS) of BMH fractions exhaustive digests (3) [ΔU(4,4,0)-2, ΔU(4,4,0)-3, ΔIIIs-IIs_glu_, ΔIs-IVs_glu_, ΔU(4,5,0)-4 to 6, ΔU(4,6,0)-7 to 9] were not quantified here. As a matter fact, if their presence is, on the structural point of view, interesting in relation to BMH specific 6-OH ATIII binding sequences, their content, estimated from LC/MS data in the first part (3) for BMH, is rather low (< 0.5%). Moreover, they were not all identified in the AS11 chromatographic system, due to its incompatibility with MS detection and its chromatographic specificity being different to ion pair chromatography. Thus, even though in some cases [ΔIIIs-IIs_glu_, ΔIs-IVs_glu_, ΔU(4,5,0)-4, and ΔU(4,6,0)-7 to 9], chromatographic specificities allowed their probable recognition, as shown in chromatograms of heparinases I+II+III digests of fractions LA, HA1, HA3, and HA5 (Figures 2–4), it is obvious that the resolving power of AS11 columns is too limited to enable their full quantification.

**TABLE 4:** Quantification of building blocks (% w/w) with heparinase I+II+III digests of PMH affinity fractions.

	PMH	Hep*	LA	HA1	HA2	HA3	HA4	HA5
	ΔIVa	1.9	2.0	1.7	1.7	1.6	1.6	1.7
	ΔIVs_gal_	0.1	0.1	0.1	0.1	0.0	0.1	0.1
	ΔIVs	2.2	2.6	1.7	1.5	1.3	1.3	1.2
	ΔIIa	2.0	2.0	1.7	1.7	1.7	2.0	1.8
	ΔIIIa	1.4	1.6	1.2	1.0	0.9	0.9	0.8
	ΔIIs_gal_	0.2	0.4	0.5	0.4	0.4	0.4	0.4
**Unsaturated building blocks**	ΔIIs	8.2	8.8	8.1	7.6	6.7	7.0	6.7
	ΔIIIs	6.1	7.1	5.5	4.6	3.9	3.8	3.6
	ΔIa	1.4	1.2	1.5	1.4	1.3	1.4	1.3
	ΔIIa-IVs_glu_	1.0	0.4	1.5	1.5	1.6	1.6	1.8
	ΔIIs	0.0	0.0	0.0	0.0	0.2	0.4	0.2
	ΔIIIs	0.0	0.0	0.0	0.0	0.1	0.0	0.0
	ΔIs	61.4	63.1	61.7	61.4	59.3	57.4	55.2
	ΔIIa-IIs_glu_	3.7	1.1	5.2	6.8	8.7	8.5	9.0
	ΔIs	0.3	0.1	0.3	0.3	0.4	0.8	1.3
	ΔIs-IdoA(2S)	0.1	0.3	0.2	0.2	0.2	0.2	0.2
	ΔIIs-IIs_glu_	0.5	0.1	0.4	0.5	1.1	2.1	3.4
	ΔIa-IIs_glu_	0.3	0.1	0.3	0.5	0.6	0.6	0.5
	ΔIs-IIs_glu_	0.4	0.1	0.4	0.6	0.9	0.7	1.0

	GlcNS	0.2	0.2	0.1	0.1	0.1	0.1	0.1
	GlcN(NS,3S)	0.1	0.1	0.1	0.1	0.1	0.1	0.1
	GlcN(NS,6S)	0.7	0.5	0.4	0.3	0.4	0.4	0.4
**NRE**	U(2,2,0)	0.4	0.4	0.4	0.4	0.4	0.3	0.3
	IIs_glu_	1.0	0.8	1.1	1.1	1.1	1.2	1.3
	Is_id_	0.7	0.9	0.8	0.7	0.6	0.6	0.7
	GlcN(NS,3S,6S)	0.3	0.3	0.4	0.5	0.5	0.4	0.5

	Sulfates/Carboxylates	2.52	2.51	2.54	2.54	2.55	2.54	2.55
	Sulfates/Carboxylates (sites)	2.09		2.05	2.07	2.11	2.18	2.24
	
	NAc (sites)	42.0		44.2	43.7	41.9	37.5	34.1
	NAc	11.7	10.2	11.9	12.7	13.5	13.8	13.5
**Heparin**	6-OH	15.4	17.4	13.9	12.5	11.2	11.1	10.8
	2-OH	25.1	22.5	26.3	27.7	29.3	31.3	32.4
	3S	4.9	2.4	6.3	7.6	9.2	10.4	11.9

	Tetra 3S/Di 3S (mole/mole)	14.6	8.8	13.4	15.5	12.8	6.7	6.1
	Sites/Chain		0.2	1.2	1.5	1.8	1.9	2.1
								

*NAc, % N-acetylated glucosamines; 6-OH, % 6-OH glucosamines; 2-OH, % 2-OH uronic acids; 3S, % 3-O-sulfated glucosamines.*

HA, high affinity; Hep*, starting heparin; LA, low affinity; PMH, porcine mucosa heparin. Tetra 3S/Di 3S: Ratio of 3S building blocks between tetrasaccharides and disaccharides, Sites/Chain: number of ATIII binding pentasaccharides by chain.

**TABLE 5:** Quantification of building blocks (% w/w) with heparinase I+II+III digests of BMH affinity fractions.

	BMH	Hep*	LA	HA1	HA2	HA3	HA4	HA5
	ΔIVa	2.1	2.3	2.1	2.0	2.0	2.0	2.0
	ΔIVs_gal_	0.2	0.3	0.2	0.2	0.2	0.1	0.1
	ΔIVs	3.7	4.1	3.2	2.9	2.5	2.5	2.2
	ΔIIa	0.5	0.5	0.5	0.5	0.5	0.6	0.7
	ΔIIIa	1.6	1.7	1.6	1.5	1.4	1.3	1.2
	ΔIIs_gal_	0.3	0.3	0.3	0.3	0.3	0.3	0.3
**Unsaturated building blocks**	ΔIIs	7.1	7.1	7.4	7.2	6.4	6.9	6.5
	ΔIIIs	23.4	26.1	22.7	17.6	14.3	13.7	13.0
	ΔIa	0.2	0.2	0.1	0.3	0.3	0.3	0.4
	ΔIIa-IVs_glu_	1.1	0.2	1.9	2.6	2.6	2.9	2.9
	ΔIIs	0.1	0.0	0.1	0.2	0.3	0.3	0.4
	ΔIIIs	0.9	0.9	1.3	1.0	0.9	1.0	0.9
	ΔIs	45.7	46.0	44.3	48.8	49.8	47.7	44.3
	ΔIIa-IIs_glu_	1.1	0.5	1.5	2.0	2.4	3.0	3.6
	ΔIs	1.1	0.7	1.6	1.4	1.5	2.3	4.2
	ΔIs-IdoA(2S)	0.2	0.2	0.1	0.1	0.2	0.1	0.3
	ΔIIs-IIs_glu_	0.4	0.0	0.5	0.7	1.0	2.1	2.6
	ΔIa-IIs_glu_	0.1	0.0	0.1	0.2	0.2	0.3	0.3
	ΔIs-IIs_glu_	0.9	0.1	1.2	1.8	3.7	3.1	2.4

	GlcNS	0.1	0.2	0.1	0.1	0.1	0.1	0.1
	GlcN(NS,3S)	0.2	0.2	0.1	0.1	0.2	0.1	0.1
	GlcN(NS,6S)	0.3	0.2	0.3	0.2	0.3	0.2	0.2
**NRE**	U(2,2,0)	0.5	0.7	0.4	0.3	0.3	0.3	0.5
	IIs_glu_	0.0	0.0	0.1	0.1	0.1	0.1	0.1
	Is_id_	2.0	2.1	1.7	1.7	1.9	1.6	1.6
	GlcN(NS,3S,6S)	0.3	0.3	0.5	0.4	0.4	0.4	0.5

	Sulfates/Carboxylates	2.36	2.33	2.37	2.41	2.47	2.47	2.48
	Sulfates/Carboxylates (sites)	2.58		2.51	2.41	2.48	2.50	2.57
	
	NAc (sites)	20.9		22.8	25.4	22.3	22.0	22.4
	NAc	7.3	6.9	7.1	8.7	9.3	9.5	10.3
**Heparin**	6-OH	38.2	40.9	37.0	31.3	27.1	26.5	25.7
	2-OH	20.9	19.0	22.0	23.3	23.8	26.2	27.5
	3S	4.6	2.6	6.2	7.0	8.6	10.4	13.4

	Tetra 3S/Di 3S (mole/mole)	1.1	0.4	1.2	1.8	2.4	2.0	1.5
	Sites/Chain	0.7	0.1	1.0	1.3	1.6	2.0	2.1
								

*NAc, % N-acetylated glucosamines; 6-OH, % 6-OH glucosamines; 2-OH, % 2-OH uronic acids; 3S, % 3-O-sulfated glucosamines.*

HA, high affinity; Hep*, starting heparin; LA, low affinity; BMH, bovine mucosa heparin. Tetra 3S/Di 3S: Ratio of 3S building blocks between tetrasaccharides and disaccharides, Sites/Chain: number of ATIII binding pentasaccharides by chain.

**TABLE 6:** Quantification of building blocks (% w/w) with heparinase I+II+III digests of BLH affinity fractions.

	BLH	Hep*	LA	HA1	HA2	HA3	HA4	HA5
	ΔIVa	0.7	0.6	0.8	0.7	0.6	0.6	0.7
	ΔIVs_gal_	0.0	0.0	0.0	0.0	0.0	0.0	0.0
	ΔIVs	0.5	0.4	0.5	0.4	0.3	0.4	0.4
	ΔIIa	0.3	0.2	0.2	0.2	0.2	0.2	0.3
	ΔIIIa	0.0	0.1	0.1	0.1	0.1	0.1	0.1
	ΔIIs_gal_	0.0	0.1	0.1	0.1	0.1	0.1	0.1
**Unsaturated building blocks**	ΔIIs	4.2	4.8	5.8	5.2	4.3	5.0	5.5
	ΔIIIs	4.8	5.1	6.3	4.7	3.8	3.7	4.0
	ΔIa	0.0	0.0	0.0	0.0	0.0	0.0	0.1
	ΔIIa-IVs_glu_	0.1	0.0	0.2	0.3	0.2	0.3	0.3
	ΔIIs	0.3	0.0	0.2	0.2	0.3	0.7	1.0
	ΔIIIs	0.1	0.0	0.4	0.2	0.1	0.2	0.2
	ΔIs	76.0	78.2	70.5	71.8	69.6	65.3	63.7
	ΔIIa-IIs_glu_	0.8	0.1	1.4	1.9	1.8	1.9	2.4
	ΔIs	0.9	0.4	1.8	1.0	1.0	1.4	3.1
	ΔIs-IdoA(2S)	0.6	0.7	0.4	0.3	0.5	0.5	0.5
	ΔIIs-IIs_glu_	0.8	0.0	1.1	1.6	2.8	4.9	5.2
	ΔIa-IIs_glu_	0.0	0.0	0.1	0.0	0.0	0.0	0.0
	ΔIs-IIs_glu_	1.3	0.1	1.2	2.8	5.3	4.9	2.8

	GlcNS	0.0	0.0	0.1	0.0	0.0	0.0	0.0
	GlcN(NS,3S)	0.2	0.2	0.2	0.2	0.2	0.2	0.2
	GlcN(NS,6S)	0.3	0.2	0.2	0.2	0.2	0.2	0.2
**NRE**	U(2,2,0)	0.1	0.2	0.2	0.1	0.1	0.1	0.1
	IIs_glu_	0.0	0.0	0	0	0	0	0
	Is_id_	3.7	4.2	2.3	2.4	3.0	2.3	2.2
	GlcN(NS,3S,6S)	0.7	0.7	0.7	0.6	0.7	0.7	0.8

	Sulfates/Carboxylates	2.84	2.84	2.80	2.79	2.83	2.82	2.81
	Sulfates/Carboxylates (sites)	2.84		2.84	2.67	2.81	2.72	2.78
	
	NAc (sites)	11.2		14.7	15.1	12.8	8.5	10.1
	NAc	2.1	1.6	2.6	2.7	2.5	2.7	3.3
**Heparin**	6-OH	7.7	9.1	10.1	7.9	7.7	6.6	7.2
	2-OH	10.0	7.7	13.0	13.9	17.6	18.6	19.7
	3S	4.0	1.8	5.1	6.7	8.0	9.9	11.2

	Tetra 3S/Di 3S (mole/mole)	1.3	0.5	1.0	2.8	4.3	3.2	1.5
	
	Sites/Chain	0.3	0.0	0.6	0.9	1.2	1.6	1.4
								

*NAc, % N-acetylated glucosamines; 6-OH, % 6-OH glucosamines; 2-OH, % 2-OH uronic acids; 3S, % 3-O-sulfated glucosamines.*

HA, high affinity; Hep*, starting heparin; LA, low affinity; BLH, bovine lung heparin. Tetra 3S/Di 3S: Ratio of 3S building blocks between tetrasaccharides and disaccharides, Sites/Chain: number of ATIII binding pentasaccharides by chain.

**FIGURE 5:**
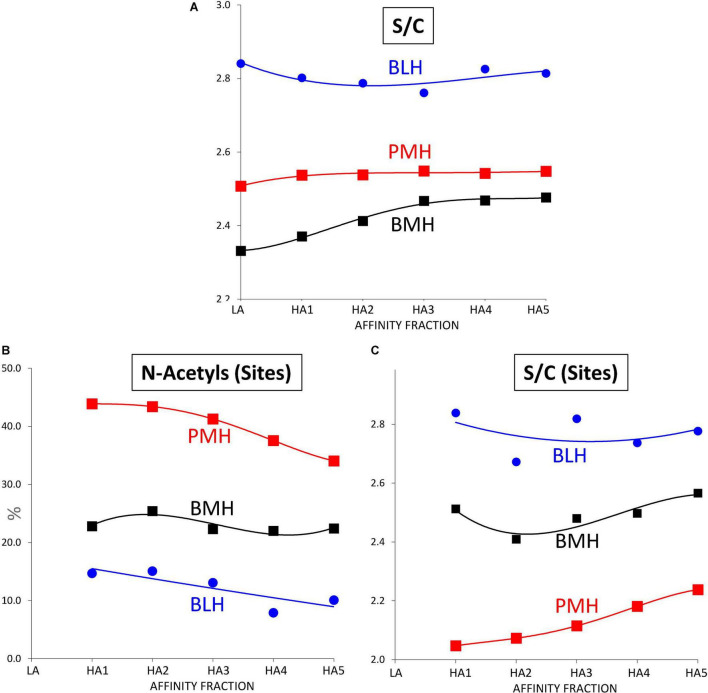
Influence of the ATIII affinity on the sulfation pattern of heparin fractions determined from building block analyses after digestion by heparinases I+II+III. **(A)** Sulfates/Carboxylates (S/C). **(B)** % of *N-*acetylated glucosamines in 3-*O* sulfated building blocks (*N-*Acetyls (Sites)). **(C)** Sulfates/Carboxylates in 3-*O* sulfated building blocks (S/C (Sites)). 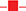
Porcine mucosa heparin (PMH); 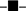
Bovine mucosa heparin (BMH); 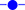
Bovine lung heparin (BLH).

Sulfate to carboxylate ratios in Tables 4–6 were determined using cumulative contributions for each building block of carboxyl and sulfate groups in the heparin. A specific determination of sulfate to carboxylate ratio and NAc% based on the same calculation with only 3S building blocks [Sulfates/Carboxylates (sites) and NAc (sites)] was also performed, as it has good specificity to the heparin source. This specificity clearly appears in Figure 5 where the variations of these three parameters were plotted against the affinity for ATIII of the fractions. In PMH, NAc (sites) had an average value of 42 (42% of disaccharides in 3S building blocks are acetylated) which means that 84% of ATIII pentasaccharide binding sites were acetylated, if the major tetrasaccharide structure of 3S building blocks is considered. In the case of BMH, NAc (sites) is lower than in PMH, usually at about 19–23, in part due to the high content of 3S disaccharides (all *N*-sulfated and included in the calculation). If only 3S tetrasaccharides were considered, values at about 30 would be obtained, to be compared with the 42 obtained for PMH. With Sulfates/Carboxylates (sites), these two parameters reflect the differences of sulfate distribution in the ATIII binding sites for each heparin source.

**FIGURE 6:**
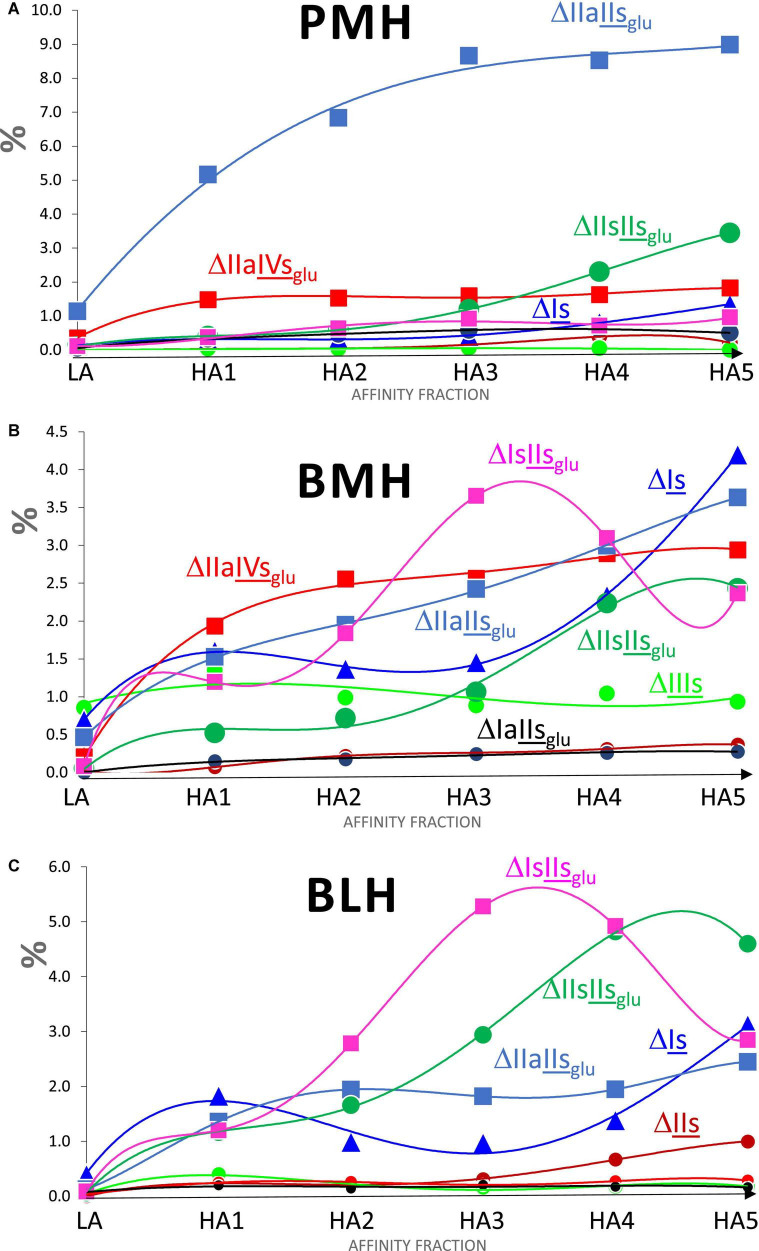
Influence of the ATIII affinity on the percentages (w/w) of 3-*O* sulfated building blocks found in heparins fractions digested by heparinase I+II+III. **(A)** Porcine mucosa heparin (PMH). **(B)** Bovine mucosa heparin (BMH). **(C)** Bovine lung heparin (BLH); Building block: 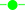
ΔIIIs; 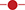
ΔIIs; 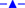
ΔIs; 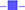
ΔIIaIIs_glu_;
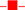
ΔIIaIVs_glu_; 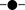
ΔIaIIs_glu_;
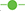
ΔIIsIIs_glu_; 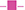
ΔIsIIs_glu_.

Increased affinity of the fraction was associated with both an increase in the number of ATIII binding sites per chain and a structural transformation of these binding sites. The number of sites per chain was estimated from the content of the five major 3S tetrasaccharides (ΔIIa-IVs_glu_, ΔIIa-IIs_glu_, ΔIIs-IIs_glu_, ΔIa-IIs_glu_, and ΔIs-IIs_glu_), based on the assumption that theses tetrasaccharides were all digested from true ATIII binding pentasaccharides. Values greater than two were obtained from fractions of highest affinity, even in BMH, although the calculation did not consider the influence of 3S disaccharides. The degree of 3-*O*-sulfation, which follows a similar increasing trend, offers another way to detect the same phenomenon. The structure-activity relationships governing the binding of pentasaccharides sites to ATIII are common to all heparins, so that the structural transformation of binding sites observed in fractions of increasing affinity to ATIII had similarities between the three heparins, but there were however significant specific differences, particularly for BMH, that highly impacted this transformation. Figure 6 shows the variations for PMH, BMH and BLH of the percentages of the main 3-*O* sulfated building blocks when the affinity of the fraction for ATIII increases. For PMH and BLH, NAc (sites) decreased between HA1 and HA5 (Figure 5B), reflecting the smaller growth of acetylated building blocks ΔIIa-IVs_glu_ and ΔIIa-IIs_glu_ compared with the *N*-sulfated blocks, ΔIs-IIs_glu_ and ΔIIs-IIs_glu_. Doubling of AXa activity induced by the substitution of NAc for NS in the non-reducing glucosamine of ATIII binding pentasaccharides has previously been observed (14, 15). ΔIIs-IIs_glu_ appears to generate pentasaccharides with highest affinity to ATIII since it is the building block where the concentration increase between fractions HA1 to HA5, reaches highest values, to a much greater extent than for ΔIs-IIs_glu_. We have already observed in high-affinity hexa- and octasaccharides from Semuloparin (10) that the 2-*O*-sulfate of ΔIs-IIs_glu_- induced a marked decrease of affinity for ATIII binding sites, illustrated by the stronger binding to the ATIII affinity chromatography stationary phase of the hexasaccharide ΔIIs-IIs_glu_-Is_id_ than the Δ2-*O*-sulfated equivalent ΔIs-IIs_glu_-Is_id_. The strong increases of ΔIs, observed for bovine heparins, will be discussed later with other 3S disaccharides.

**FIGURE 7:**
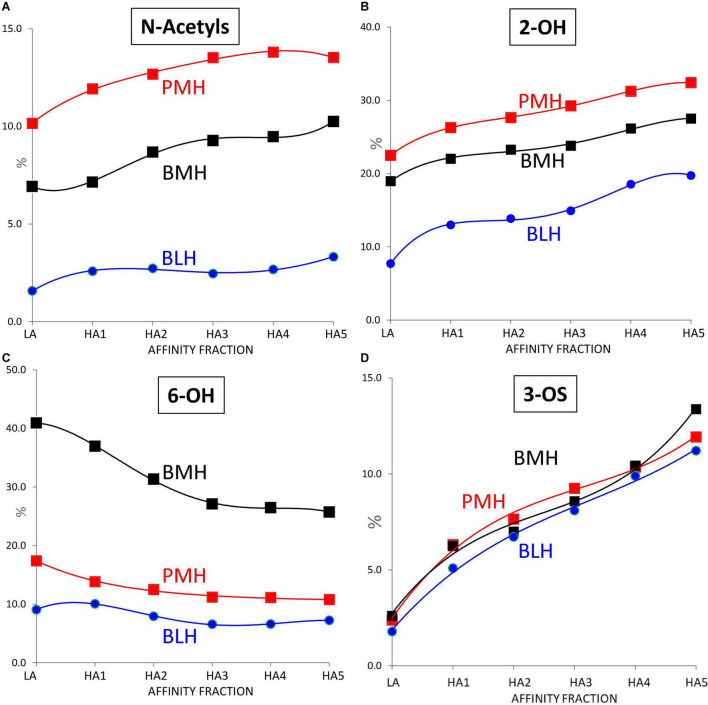
Influence of the ATIII affinity on the sulfation pattern of heparin fractions determined from building block analyses after digestion by heparinases I+II+III. **(A)** % of *N*-acetylated glucosamines. **(B)** % of 2-OH uronic acids. **(C)** % of 6-OH glucosamines. **(D)** % of 3-*O* sulfated glucosamines. 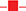
Porcine mucosa heparin (PMH); 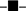
Bovine mucosa heparin (BMH); 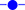
Bovine lung heparin (BLH).

Some other general trends were detected in Figure 7 where the variation of the parameters reflecting the sulfation pattern of the heparin fractions were plotted against the ATIII affinity of the fraction: with increasing affinity for ATIII, the percentage of 6-*O*-sulfation increased (Figure 7C), and 2-*O*-sulfation decreased (Figure 7B), which can largely be explained by the increase in ATIII binding sites (one 3-*O*-sulfate and one 2-OH GlcA), and the higher affinity of their 6-*O*-sulfated derivatives.

Other specific patterns were detected, such as the presence of the NRE PMH marker IIs_glu_^sulf^ in fractions of PMH (7) (Table 4 and Supplementary Figures 1–8). Another specificity observed for BMH (Table 5) was the increase of the sulfate to carboxylate ratio of fractions with their affinity for ATIII (Figure 5A). This small but significant difference, only observed with BMH, was probably the reason why fractions with higher anticoagulant activity could be obtained from BMH by anion exchange chromatography (16).

However, despite all differences observed in Figures 5–7 between the 3 heparin origins, the similar trend of the percentages of 3-*O* sulfated glucosamines with the affinity for ATIII, observed in Figure 7D, is an indicator of a good comparability of the affinity chromatography experiments performed on the three heparins.

#### 3S Disaccharides

The distribution of 3S disaccharides in the affinity fractions LA and HA1–HA5 was central to ascertaining their role in binding to ATIII. ΔIIs, which was only present in the HA fractions of the three heparin sources and at a content increasing with the fraction affinity (Figure 6), must be classified as ATIII-binding. It strengthens the option chosen in the first part (3) of IIs_glu_ as precursor of ΔIIs within structural sequences with consecutive 3S disaccharides. Figure 6 and the data from Tables 4–6 also show that the content of ΔIs was clearly associated with the affinity of the fraction for ATIII, which reaches significant levels mainly in bovine heparins (Figures 6B,C) and, to a lesser extent, in ovine mucosa heparin (7).

The molar ratio within 3S building blocks between tetrasaccharides and disaccharides is given in Tables 4–6. This ratio increased with increasing affinity to reach a maximum for HA2 or HA3 for each type of heparin. High values were obtained with PMH due to the low content of 3S disaccharides. Much lower values were obtained for bovine heparins but in BLH, the 3S disaccharides were mainly ΔIs and ΔIIs, found in the high-affinity fractions, while in BMH, ΔIs and ΔIIIs were both present, in line with the low 6-*O*-sulfation of this heparin.

The variations of ΔIs in Figure 6 show for the three heparin origins, a more pronounced increase of the ΔIs content was observed for fractions of highest ATIII affinity (HA3–HA5), due to the presence in these fractions of ATIII binding sites with a supplementary 3-*O*-sulfate such as -IIa_id_-IIs_glu_-Is_id_-, which have a higher affinity for ATIII than conventional binding pentasaccharides (3, 17, 18). ΔIIa-IIs_glu_-Is_id_ is heparinase II-resistant but was digested into ΔIIa-IIs_glu_ and ΔIs by the heparinase mixture. These sequences were obtained by a secondary reaction of 3-*O*-sulfotransferases on the last glucosamine of the pentasaccharide. However, ΔIs was also detected in HA1, HA2, HA3 and partial LA fractions where the double 3-*O*-sulfated ATIII binding pentasaccharides were not present.

The main precursors of ΔIs, found in the corresponding heparinase II digests, were the two tetrasaccharides ΔIIs-Is_id_ and ΔIs-Is_id_. The sequences -IIs_glu_-Is_id_-Is_id_ [-GlcA-GlcN(NS,6S)-IdoA(2S)-GlcN(NS,3S,6S)-IdoA(2S)-GlcN(NS,6S)-) and -Is_id_-Is_id_-Is_id_ (-IdoA(2S)-GlcN(NS,6S)-IdoA(2S)-GlcN(NS,3S,6S)-IdoA(2S)-GlcN(NS,6S)-] with potential affinity for ATIII can be hypothesized since significant affinity for ATIII and AXa activity were measured for the biosynthetic octasaccharide GlcN(NS,6S)-GlcA-GlcN(NS,6S)-IdoA(2S)-GlcN(NS,3S,6S)-IdoA(2S)-GlcN(NS,6S)-GlcA (5).

The presence of ΔIs in the LA fraction could be used as an argument against the affinity of sequences including Is_id_. However, the LA fraction of PMH also contained some ΔIIa-IIs_glu_ and had a residual activity (Table 3). Indeed, the selectivity of ATIII affinity chromatography between HA and LA fractions had an inverse relationship to molecular weight. The BLH batch used for this analysis is much smaller than the PMH and BMH batches (Table 2), so that the LA fraction of BLH contains very little residual 3S tetrasaccharides (Table 6), with the ratio of the percentages of ΔIs between HA1 and LA almost twice that obtained for BMH (Table 5). If we consider a LMWH synthesized from BMH using enoxaparin process (unpublished data), the content of ΔIs in the high affinity fraction is more than twice that in the starting heparin when a residual 0.5% is detected in the LA fraction. When the length of the chain is decreased, the structural specificity of fractions increases so that, for hexasaccharides, the percentages of ΔIs in HA and LA are 8.1% and 0.4%, respectively. As shown in Figure 6, particularly in the case of BMH (Figure 6B), the case of ΔIIIs is less clear, with desulfation in position 6-*O* probably responsible for the lower affinity of sequences. Unlike ΔIs, there was no indication of any double 3S ATIII pentasaccharides ending with IIIs_id_. Moreover, the same shared presence of ΔIIIs as for ΔIs was observed with LA and HA, but with a higher part in LA, particularly in the case of BMH. In bovine LMWH, as for ΔIs, ΔIIIs is still more concentrated in HA fractions (2.1% vs. 0.5% for LA). But finally, it seems that the affinity of sequences containing IIIs for ATIII should be at least low if not totally negligible.

#### Heparinase II Digestion of Heparin Affinity Fractions

In the first part of these experiments (3), heparinase II digests of BMH fractions were studied to highlight new specific 3S tetrasaccharide and hexasaccharide building blocks cleaved into unsaturated disaccharides (ΔIs, ΔIIs, and ΔIIIs) in exhaustive digests, by heparinase I. Nine new tetrasaccharides [ΔU(4,5,0)-10 to 13, ΔU(4,6,0)-14 to 16, ΔIIs-Is_id_, ΔIs-Is_id_] were detected, and most were specific to BMH. ΔU(4,6,0)-16 was also present in BLH while ΔIIs-Is_id_ and ΔIs-Is_id_ were both present at least in PMH, BLH and ovine mucosa heparin (OMH). Hexasaccharides were mainly detected in fractions of highest affinity (HA4 and HA5), mainly ΔIIa-IVs_glu_-Is_id_, ΔIIa-IIs_glu_-Is_id_, ΔIa-IIs_glu_-Is_id_, ΔIIs-IIs_glu_-Is_id_, and ΔIs-IIs_glu_-Is_id_, with other hexasaccharides as yet unidentified.

**FIGURE 8:**
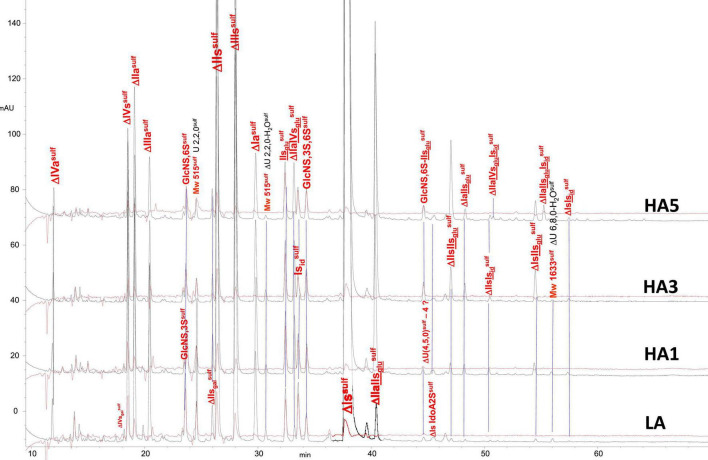
Chromatogram on AS11 of heparinase II digests from porcine mucosa heparin (PMH) affinity fractions with sulfanilic tagging. Detection: (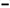) 265 nm; (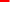) 265 nm – 2.21 × 232 nm (UV selective saturated NRE signal).

**FIGURE 9:**
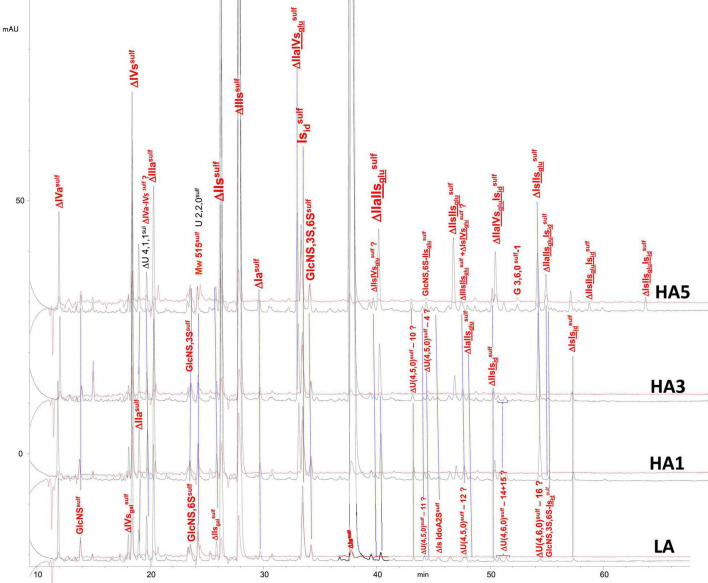
Chromatogram on AS11 of heparinase II digests from bovine mucosa heparin (BMH) affinity fractions with sulfanilic tagging. Detection: (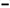) 265 nm; (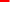) 265 nm – 2.21 × 232 nm (UV selective saturated NRE signal).

**FIGURE 10:**
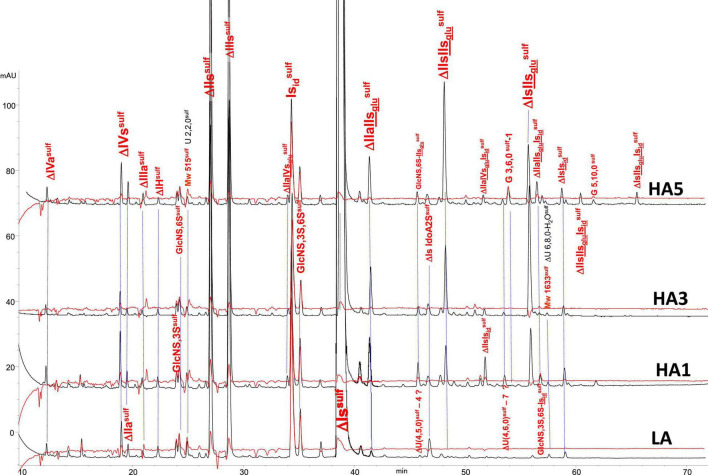
Chromatogram on AS11 of heparinase II digests from bovine lung heparin (BLH) affinity fractions with sulfanilic tagging. Detection: (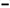) 265 nm; (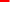) 265 nm – 2.21 × 232 nm (UV selective saturated NRE signal).

Integration data obtained from AS11 chromatograms of the heparinase II digests from PMH, BMH and BLH (Figures 8–10) are shown in Tables 7–9. As done for the heparinase I+II+III digests, the influence of the heparin origin on the separation on AS11 column of the building blocks obtained from heparinase II digestion of the different affinity fractions (LA, HA1, HA3 and HA5) is shown is the Supplementary Figures 5–8. Additionally, the influence of the digestion type (Heparinases I+II+III vs. Heparinase II) for each type of affinity fraction and for one animal origin of heparin is also illustrated in the Supplementary Figures 9–21. As observed in the heparinases I+II+III digests, some of the heparinase II specific building blocks detected in the ion pair LC/MS could be identified in the AS11 chromatographic system. However, Tables 7–9 only include the w/w% of the main building blocks with the already ascertained heparinase II-specific ones, ΔIIs-Is_id_, ΔIs-Is_id_, ΔIIa-IVs_glu_-Is_id_, ΔIIa-IIs_glu_-Is_id_, ΔIa-IIs_glu_-Is_*i*__*d*_, ΔIIs-IIs_glu_-Is_id_, and ΔIs-IIs_glu_-Is_id_. Sulfate to carboxylate ratios and percentages (NAc, 2-OH, 6-OH, 3S)% were not given, because the *N*-acetyl and 3S building blocks could not be exhaustively identified due to their scattering into unidentified glycoserines and other minor heparinase II-specific building blocks. Overall, aside from ΔIVa, ΔIVs and 3S building blocks, integration data from heparinase II and heparinases I+II+III were similar. In the three heparins (Tables 7–9), ΔIIs-Is_id_ and ΔIs-Is_id_ were major fragments containing -Is_id_ and their presence, detected essentially in the affinity fractions, was a strong argument for their ATIII binding contribution within a non-conventional pentasaccharide where a 2-*O*-sulfated iduronic acid is substituted for the canonical central glucuronic acid. These data, compatible with an ATIII binding capacity of sequences including ΔIIs-Is_id_ and ΔIs-Is_id_, are in line with those recently published (5, 6). Table 7 also shows that even in the fraction HA5, the phenomenon of double 3S ATIII pentasaccharides was limited for PMH (5–10% of ΔIIa-IVs_glu_, and ΔIIa-IIs_glu_ is in ΔIIa-IVs_glu_-Is_id_, ΔIIa-IIs_glu_-Is_id_ sequences) in connection with the low content of ΔIs. Much higher percentages (10–40%) were observed in BMH (Table 8) and in BLH. The sites present in double 3S sequences reflected similar sulfation patterns than the classical ATIII pentasaccharides detected in lower affinity fractions, supporting the idea that this effect is due to a secondary reaction of 3-*O*-sulfotransferases after the first 3-*O*-sulfation of the site, key element of the pentasaccharide anticoagulant activity. However, maximum percentages (40%) were observed in acetylated sites (ΔIIa-IVs_glu_-Is_id_, ΔIIa-IIs_glu_-Is_id_).

**TABLE 7:** Quantification of building blocks (% w/w) with heparinase II digests of PMH affinity fractions.

	PMH	Hep*	LA	HA1	HA2	HA3	HA4	HA5
	ΔIVa	0.2	0.2	0.2	0.1	0.2	0.3	0.2
	ΔIVs_gal_	0.1	0.1	0.1	0.1	0.1	0.1	0.1
	ΔIVs	1.8	2.2	1.5	1.3	1.1	1.2	0.9
	ΔIIa	1.8	2.0	1.7	1.6	1.5	1.7	1.6
	ΔIIIa	1.3	1.6	1.1	1.0	0.9	0.9	0.8
	ΔIIs_gal_	0.5	0.4	0.5	0.4	0.4	0.4	0.5
**Unsaturated building blocks**	ΔIIs	8.5	9.3	8.1	7.6	6.9	7.0	6.7
	ΔIIIs	6.3	7.4	5.5	4.6	3.9	3.8	3.7
	ΔIa	1.4	1.3	1.5	1.4	1.3	1.3	1.4
	ΔIIa-IVs_glu_	0.9	0.4	1.5	1.5	1.6	1.6	1.8
	ΔIIs	0.0	0.0	0.0	0.0	0.0	0.0	0.0
	ΔIIIs	0.0	0	0	0.0	0.0	0.0	0.0
	ΔIs	63.6	65.3	62.9	62.3	61.0	58.7	56.7
	ΔIIa-IIs_glu_	3.6	1.1	5.3	6.9	8.9	8.4	8.4
	ΔIs	0.0	0.0	0.0	0.0	0.0	0.0	0.0
	ΔIs-IdoA(2S)	0.1	0.2	0.2	0.2	0.2	0.3	0.3
	ΔIIs-IIs_glu_	0.5	0.1	0.4	0.6	1.2	2.3	3.4
	ΔIa-IIs_glu_	0.3	0.1	0.4	0.6	0.7	0.6	0.5
	ΔIIs-Is_id_	0.1	0.0	0.2	0.2	0.2	0.2	0.2
	ΔIIa-IVs_glu_-Is_id_						0.0	0.2
	ΔIs-IIs_glu_	0.4	0.1	0.5	0.9	1.2	1.0	0.9
	ΔIIa-IIs_glu_-Is_id_						0.2	0.8
	ΔIs-Is_id_	0.1	0.0	0.1	0.1	0.1	0.2	0.2
	ΔIIs-IIs_glu_-Is_id_							
	ΔIs-IIs_glu_-Is_id_							

	GlcNS	0.1	0.1	0.1	0.1	0.1	0.1	0.1
	GlcN(NS,3S)	0.1	0.1	0.1	0.1	0.1	0.1	0.1
	GlcN(NS,6S)	0.6	0.6	0.4	0.4	0.4	0.4	0.4
**NRE**	U(2,2,0)	0.4	0.4	0.4	0.3	0.3	0.3	0.3
	IIs_glu_	0.9	0.9	1.1	1.1	1.1	1.1	1.2
	Is_id_	0.7	1.0	0.8	0.8	0.6	0.7	0.8
	GlcN(NS,3S,6S)	0.4	0.3	0.4	0.4	0.5	0.4	0.5

HA, high affinity; Hep*, starting heparin; LA, low affinity; PMH, porcine mucosa heparin.

**TABLE 8:** Quantification of building blocks (% w/w) with heparinase II digests of BMH affinity fractions.

	BMH	Hep*	LA	HA1	HA2	HA3	HA4	HA5
	ΔIVa	0.5	0.7	0.7	0.6	0.7	0.6	0.6
	ΔIVs_gal_	0.2	0.3	0.3	0.2	0.2	0.2	0.1
	ΔIVs	2.7	3.3	2.7	2.4	2.1	2.0	1.7
	ΔIIa	0.4	0.4	0.5	0.5	0.4	0.5	0.6
	ΔIIIa	1.5	1.7	1.6	1.4	1.3	1.3	1.3
	ΔIIs_gal_	0.3	0.3	0.3	0.3	0.3	0.3	0.3
**Unsaturated building blocks**	ΔIIs	6.9	7.1	7.0	6.8	6.0	6.4	6.1
	ΔIIIs	23.6	26.2	22.9	17.7	14.3	14.0	13.4
	ΔIa	0.3	0.2	0.3	0.3	0.3	0.3	0.4
	ΔIIa-IVs_glu_	1.0	0.2	1.9	2.6	2.6	2.8	2.4
	ΔIIs		0.0	0.0	0.0	0.0	0.0	0.1
	ΔIIIs		0.0	0.0	0.0	0.0	0.0	0.1
	ΔIs	46.9	46.8	45.1	49.9	50.2	48.5	45.8
	ΔIIa-IIs_glu_	1.0	0.5	1.6	2.0	2.4	2.6	2.5
	ΔIs	0.0	0.0	0.1	0.0	0.1	0.2	0.1
	ΔIs-IdoA(2S)	0.2	0.2	0.1	0.1	0.2	0.2	0.2
	ΔIIs-IIs_glu_	0.5	0.1	0.5	0.7	1.1	2.2	2.4
	ΔIa-IIs_glu_	0.1	0.0	0.1	0.3	0.3	0.3	0.3
	ΔIIs-Is_id_	0.3	0.1	0.7	0.5	0.5	0.6	0.7
	ΔIIa-IVs_glu_-Is_id_	0.1					0.5	2.5
	ΔIs-IIs_glu_	1.1	0.1	1.1	2.1	4.2	3.2	1.9
	ΔIIa-IIs_glu_-Is_id_	0.1					0.3	2.0
	ΔIs-Is_id_	0.3	0.2	0.3	0.3	0.4	0.6	0.7
	ΔIIs-IIs_glu_-Is_id_						0.0	0.4
	ΔIs-IIs_glu_-Is_id_						0.1	0.7

	GlcNS	0.1	0.2	0.1	0.1	0.1	0.1	0.1
	GlcN(NS,3S)	0.1	0.2	0.1	0.1	0.1	0.1	0.1
	GlcN(NS,6S)	0.3	0.2	0.2	0.2	0.2	0.2	0.2
**NRE**	U(2,2,0)	0.6	0.7	0.4	0.3	0.3	0.3	0.3
	IIs_glu_	0.0	0.0	0.0	0.0	0.1	0.1	0.1
	Is_id_	2.0	2.1	1.6	1.8	1.9	1.7	1.6
	GlcN(NS,3S,6S)	0.3	0.3	0.3	0.3	0.3	0.3	0.3
								

HA, high affinity; Hep*, starting heparin; LA, low affinity; BMH, bovine mucosa heparin.

**TABLE 9:** Quantification of building blocks (% w/w) with heparinase II digests of BLH affinity fractions.

	BLH	Hep*	LA	HA1	HA2	HA3	HA4	HA5
	ΔIVa	0.1	0.1	0.1	0.1	0.1	0.1	0.1
	ΔIVs_gal_	0.0	0.0	0.0	0.0	0.0	0.0	0.0
	ΔIVs	0.2	0.3	0.4	0.3	0.2	0.3	0.4
	ΔIIa	0.1	0.1	0.1	0.1	0.1	0.1	0.2
	ΔIIIa	0.1	0.1	0.1	0.1	0.1	0.1	0.1
	ΔIIs_gal_	0.1	0.1	0.1	0.1	0.1	0.1	0.1
**Unsaturated building blocks**	ΔIIs	4.8	4.7	5.3	4.9	3.9	4.6	5.2
	ΔIIIs	4.9	5.1	6.4	4.7	3.7	3.7	4.0
	ΔIa	0.1	0.0	0.0	0.0	0.0	0.1	0.1
	ΔIIa-IVs_glu_	0.1	0.0	0.2	0.3	0.2	0.3	0.3
	ΔIIs	0.0	0.0	0.0	0.0	0.0	0.0	0.0
	ΔIIIs	0.0	0.0	0.0	0.0	0.0	0.0	0.0
	ΔIs	76.1	78.2	71.0	72.4	69.8	66.9	66.0
	ΔIIa-IIs_glu_	0.8	0.4	1.3	2.0	1.8	1.8	1.7
	ΔIs	0.0	0.1	0.1	0.1	0.0	0.0	0.1
	ΔIs-IdoA(2S)	0.4	0.7	0.4	0.3	0.5	0.5	0.4
	ΔIIs-IIs_glu_	0.9	0.1	1.2	1.7	2.9	4.8	4.6
	ΔIa-IIs_glu_		0.0	0.0	0.0	0.0	0.0	0.0
	ΔIIs-Is_id_	0.2	0.0	0.8	0.3	0.3	0.3	0.4
	ΔIIa-IVs_glu_-Is_id_						0.0	0.1
	ΔIs-IIs_glu_	1.5	0.1	1.5	3.2	6.0	4.9	2.3
	ΔIIa-IIs_glu_-Is_id_						0.2	1.1
	ΔIs-Is_id_	0.5	0.2	0.6	0.4	0.5	0.6	0.7
	ΔIIs-IIs_glu_-Is_id_						0.0	0.6
	ΔIs-IIs_glu_-Is_id_						0.1	0.7

	GlcNS	0.0	0.0	0.0	0.0	0.0	0.1	0.0
	GlcN(NS,3S)	0.1	0.1	0.1	0.1	0.1	0.1	0.1
	GlcN(NS,6S)	0.2	0.2	0.2	0.2	0.2	0.2	0.2
**NRE**	U(2,2,0)	0.2	0.2	0.0	0.1	0.1	0.1	0.1
	IIs_glu_	0.0	0.0	0.0	0.0	0.1	0.1	0.1
	Is_id_	3.7	4.2	2.3	2.4	3.0	2.4	2.3
	GlcN(NS,3S,6S)	0.6	0.7	0.5	0.5	0.6	0.5	0.5
								

HA, high affinity; Hep*, starting heparin; LA, low affinity; BLH, bovine lung heparin.

## Conclusion

In the first part of this study (3), the unsaturated disaccharide 3S (3-*O*-sulfated) building blocks obtained from heparin after digestion by the heparinase mixture were explained by a specific cleavage by heparinase I on non-conventional 3S sequences. Thus, in the absence of heparinase I, as with the heparinase II-only digestion, the 3S sequences that were digested into 3S disaccharides with heparinases I+II+III were heparinase II-resistant on their non-reducing side, resulting in longer new building blocks. The comparison of heparinases I+II+III and heparinase II digestion was particularly interesting for bovine intestine heparin, where the high content of non-conventional 3S sequences resulted in the formation of heparinase II-resistant new building blocks, mainly tetrasaccharides but also hexasaccharides. Within heparinase II-resistant hexasaccharides generated by sequences with conjugated 3S disaccharides, ATIII binding sites with two 3-*O*-sulfates, i.e., with an extra 3-*O*-sulfate in the last glucosamine of the pentasaccharide, were detected in the fractions of highest affinity for ATIII.

In the present part of the study, heparinases I+II+III and heparinase II digestions of PMH, BMH and BLH were compared using across six fractions of increasing affinity for ATIII (LA and HA1–HA5). Building blocks were tagged by reductive amination with sulfanilic acid and the 3S building blocks detected for the three heparin sources were described and compared in relation to the affinity of the fraction. The distribution of 3S building blocks in the ATIII affinity fractions were used to examine the ATIII binding of these sequences. 3S disaccharide building blocks were present at low concentrations (<0.5% w/w) in PMH but much higher in bovine heparins (up to 50% of 3S building blocks). The heparinases I+II+III digests confirm the contribution to the binding to ATIII of the two 3S disaccharides ΔHexUA(2S))-GlcN(NS,3S,6S)(ΔIs) and ΔHexUA-GlcN(NS,3S,6S) (ΔIIs) while no particular effect was detected for the third, ΔHexUA(2S)-GlcN(NS,3S)(ΔIIIs), which was mainly present in BMH. The contents of ΔIs and ΔIIs both increased in BLH and BMH digests with increased affinity to ATIII of the fraction; in both cases ΔIs was the main 3S disaccharide present in high-affinity fractions. When the heparins were digested by heparinase II only, most of the ΔIs present in fractions of highest affinity (HA5) were involved in conjugated 3S sequences and particularly, ATIII binding sites with two 3-*O*-sulfates, as ΔHexUA-GlcNAc(6S)-GlcA-GlcN(NS,3S,6S)-IdoA(2S)-GlcN(NS,3S,6S) (ΔIIa-IIs_glu_-Is_id_). For the remaining ΔIs found in fractions of lower affinities HA1 to HA3, the two major sequences identified were ΔHexUA(2S)-GlcN(NS,6S)-IdoA(2S)-GlcN(NS,3S,6S) (ΔIs-Is_id_) and ΔHexUA-GlcN(NS,6S)-IdoA(2S)-GlcN(NS,3S,6S) (ΔIIs-Is_id_). These results are compatible with the results of a recent study (5, 6) based on biosynthetic octasaccharides, showing that the binding to ATIII remained if an iduronic 2-*O*-sulfate replaced the glucuronic acid of the conventional ATIII binding sites.

## Data Availability Statement

The original contributions presented in the study are included in the article/Supplementary Material, further inquiries can be directed to the corresponding author.

## Author Contributions

PM was responsible for all aspects of the study.

## Conflict of Interest

PM is an employee of Sanofi.

## Publisher’s Note

All claims expressed in this article are solely those of the authors and do not necessarily represent those of their affiliated organizations, or those of the publisher, the editors and the reviewers. Any product that may be evaluated in this article, or claim that may be made by its manufacturer, is not guaranteed or endorsed by the publisher.

## Abbreviations

AXa: Anti-Xa
AXa: Anti-Xa
ATIII: Antithrombin III
BSA: Bovine Serum Albumin
BLH: Bovine lung heparin
BMH: Bovine mucosal heparin
GPC: Gel Permeation Chromatography
HA: High Affinity
LA: Low Affinity
LMWH: Low-molecular-weight heparin
Mw: Molecular weight
NR: Non-reducing
NRE: Non-reducing end
NRS: Non-reducing side
PMH: Porcine mucosal heparin
Qty: Quantity
RE: Reducing end
RS: Reducing side
SAX: Strong Anion Exchange
3S: 3-*O*-sulfated.

## References

[1] Santos GRC Tovar AMF Capille NVM Pereira MS Pomin VH Mourão PAS. Structural and functional analyses of bovine and porcine intestinal heparins confirm they are different drugs. *Drug Discov Today.* (2014) 19:1801–7. 10.1016/j.drudis.2014.07.004 25019497

[2] Hogwood J Mulloy B Gray E. Precipitation and neutralization of heparin from different sources by protamine sulfate. *Pharmaceuticals.* (2017) 10:59. 10.3390/ph10030059 28671597 PMC5620603

[3] Mourier PAJ. Heparinase digestion of 3-*O*-sulfated sequences: selective heparinase II digestion for separation and identification of binding sequences present in ATIII affinity fractions of bovine intestine heparins. *Front Med.* (2022) 9:841726. 10.3389/fmed.2022.841726 PMC9009448 35433769

[4] Mochizuki H Futatsumori H Suzuki E Kimata K. A quantitative method to detect non-antithrombin-binding 3-*O*-sulfated units in heparan sulfate. *J Biol Chem.* (2020) 96:100115. 10.1074/jbc.RA120.015864 33234593 PMC7948761

[5] Wang Z Hsieh PH Xu Y Thieker D En Chai EJ Xie S Synthesis of 3-*O*-sulfated oligosaccharides to understand the relationship between structures and functions of heparan sulfate. *J Am Chem Soc.* (2017) 139:5249–56. 10.1021/jacs.7b01923 28340300 PMC5624809

[6] Elli S Stancanelli E Wang Z Petitou M Liu J Guerrini M. Degeneracy of the antithrombin binding sequence in heparin: 2-*O*-sulfated iduronic acid can replace the critical glucuronic acid. *Chemistry.* (2020) 10:11814–8. 10.1002/chem.202001346 32515841

[7] Mourier PAJ. Specific non-reducing ends in heparins from different animal origins: building blocks analysis using reductive amination tagging by sulfanilic acid. *Molecules.* (2020) 25:5553. 10.3390/molecules25235553 33256116 PMC7730200

[8] Höök M Björk I Hopwood J Lindahl U. Anticoagulant activity of heparin: separation of high-activity and low-activity heparin species by affinity chromatography on immobilized antithrombin. *FEBS Lett.* (1976) 66:90–3. 10.1016/0014-5793(76)80592-3 1278445

[9] Petitou M Casu B Lindahl U. 1976–1983, a critical period in the history of heparin: the discovery of the antithrombin binding site. *Biochimie.* (2003) 85:83–9. 10.1016/s0300-9084(03)00078-6 12765778

[10] Mourier PA Guichard OY Herman F Sizun P Viskov C. New insights in thrombin inhibition structure-activity relationships by characterization of octadecasaccharides from low molecular weight heparin. *Molecules.* (2017) 22:428. 10.3390/molecules22030428 28282887 PMC6155232

[11] Mourier PA Agut C Souaifi-Amara H Herman F Viskov C. Analytical and statistical comparability of generic enoxaparins from the US market with the originator product. *J Pharm Biomed Anal.* (2015) 115:431–42. 10.1016/j.jpba.2015.07.038 26280926

[12] Mourier PAJ Herman F Sizun P Viskov C. Analytical comparison of a US generic enoxaparin with the originator product: the focus on comparative assessment of antithrombin-binding components. *J Pharm Biomed Anal.* (2016) 129:542–50. 10.1016/j.jpba.2016.07.033 27497655

[13] Mourier P Anger P Martinez C Herman F Viskov C. Quantitative compositional analysis of heparin using exhaustive heparinase digestion and strong anion exchange chromatography. *Anal Chem Res.* (2015) 3:46–53. 10.1016/j.ancr.2014.12.001

[14] van Boeckel CAA Petitou M. The unique anti-thrombin III binding domain of heparin: a lead to new synthetic antithrombotics. *Angew Chem Int Ed Engl.* (1993) 32:1671–90. 10.1002/anie.199316713

[15] Petitou M van Boeckel CA. A synthetic antithrombin III binding pentasaccharide is now a drug! What comes next? *Angew Chem Int Ed Engl.* (2004) 43:3118–33. 10.1002/anie.200300640 15199558

[16] Tovar AMF Vairo BC Oliveira SMCG Glauser BF Santos GRC Capillé N Converting the distinct heparins sourced from bovine or porcine mucosa into a single anticoagulant drug. *Thromb Haemost.* (2019) 119:618–32. 10.1055/s-0039-1678663 30791055

[17] Guerrini M Elli S Mourier P Rudd TR Gaudesi D Casu B An unusual antithrombin-binding heparin octasaccharide with an additional 3-O-sulfated glucosamine in the active pentasaccharide sequence. *Biochem J.* (2013) 449:343–51. 10.1042/BJ20121309 23083208

[18] van Boeckel CAA Beetz T van Aelst SF. Synthesis of a potent antithrombin activating pentasaccharide: a new heparin-like fragment containing two 3-O-sulphated glucosamines. *Tetrahedron Lett.* (1988) 29:803–6. 10.1016/S0040-4039(00)80214-2

