# Supplementary material for: Building Block Analysis of ATIII Affinity Fractions of Heparins: Application to the ATIII Binding Capacity of Non-conventional 3-O-Sulfated Sequences
Source: Front Med (Lausanne). 2022 Apr 19;9:841738. doi: 10.3389/fmed.2022.841738 (PMC9063521; doi:10.3389/fmed.2022.841738)
Supplement: Supplementary file 1 [file Data_Sheet_1.pdf]

Supplementary Material

Building block analysis of ATIII affinity fractions of heparins: application to the ATIII binding capacity of non-conventional 3-0-sulfated sequences

1 Influence of the heparin origin on the digests of ATIII affinity fractions

1.1 Digests by heparinase I+II+III

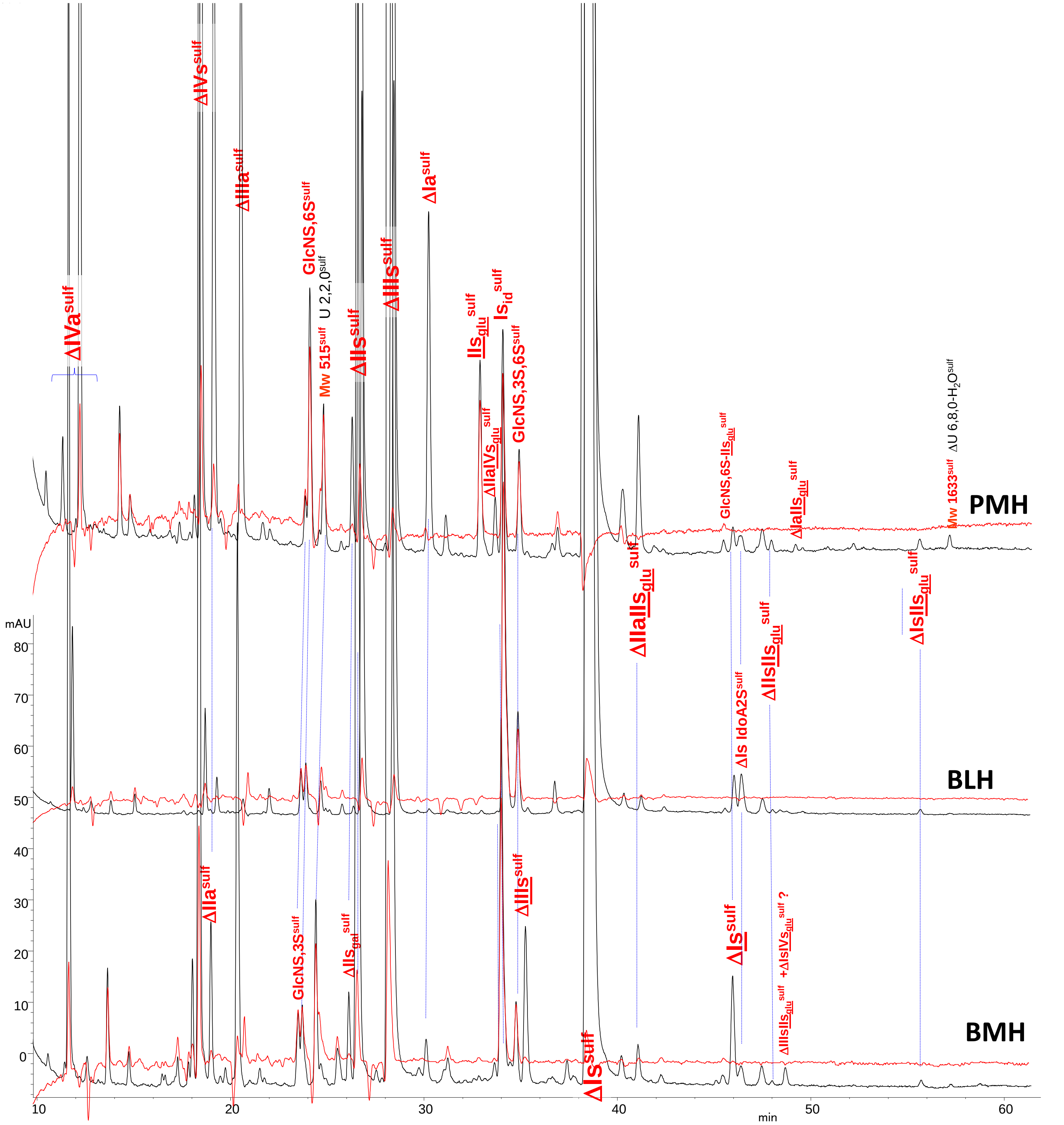

S-Figure 1: Chromatograms on AS11 of exhaustive heparinase digests of the ATIII low affinity (LA) fractions from 3 heparin sources (PMH, BLH, BMH) with sulfanilic tagging. Detection: — 265 nm; — 265 nm - 2.21 x 232 nm (UV selective saturated NRE signal).

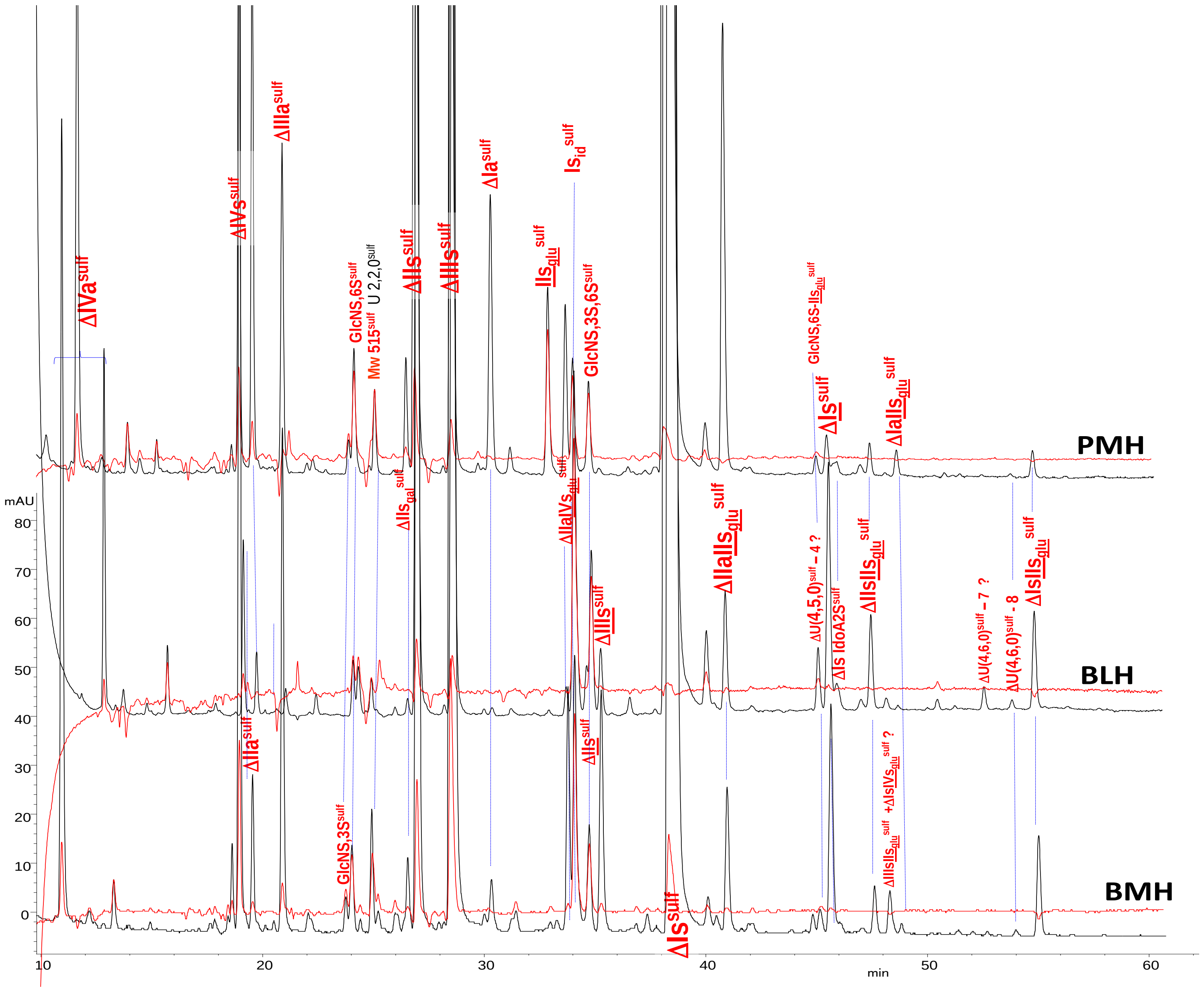

S-Figure 2: Chromatograms on AS11 of exhaustive heparinase digests of the ATIII high affinity fractions 1 (HA1) from 3 heparin sources (PMH, BLH, BMH) with sulfanilic tagging. Detection: — 265 nm; — 265 nm - 2.21 x 232 nm (UV selective saturated NRE signal).

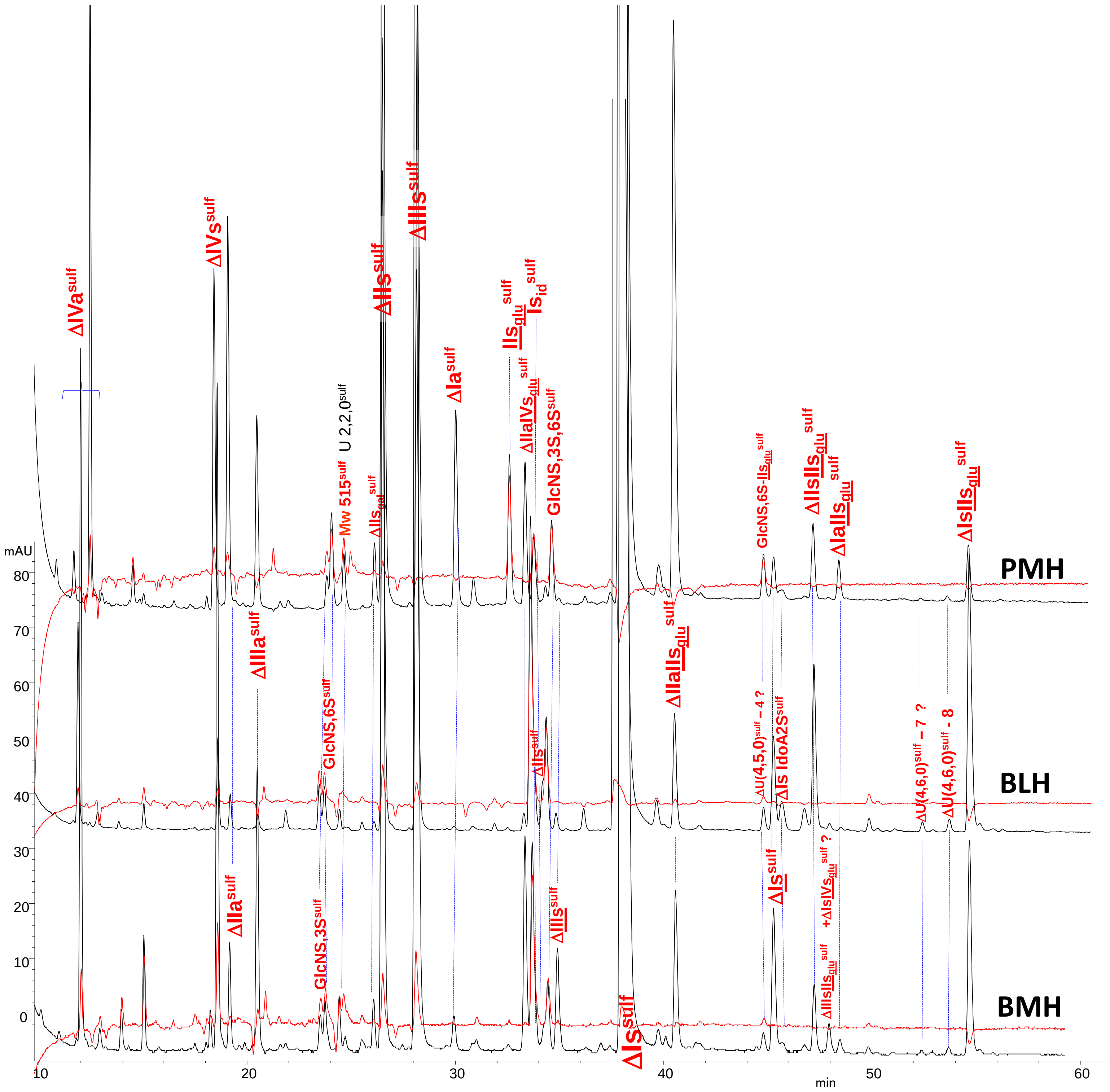

S-Figure 3: Chromatograms on AS11 of exhaustive heparinase digests of the ATIII high affinity fractions 3 (HA3) from 3 heparin sources (PMH, BLH, BMH) with sulfanilic tagging. Detection: — 265 nm; — 265 nm - 2.21 x 232 nm (UV selective saturated NRE signal).

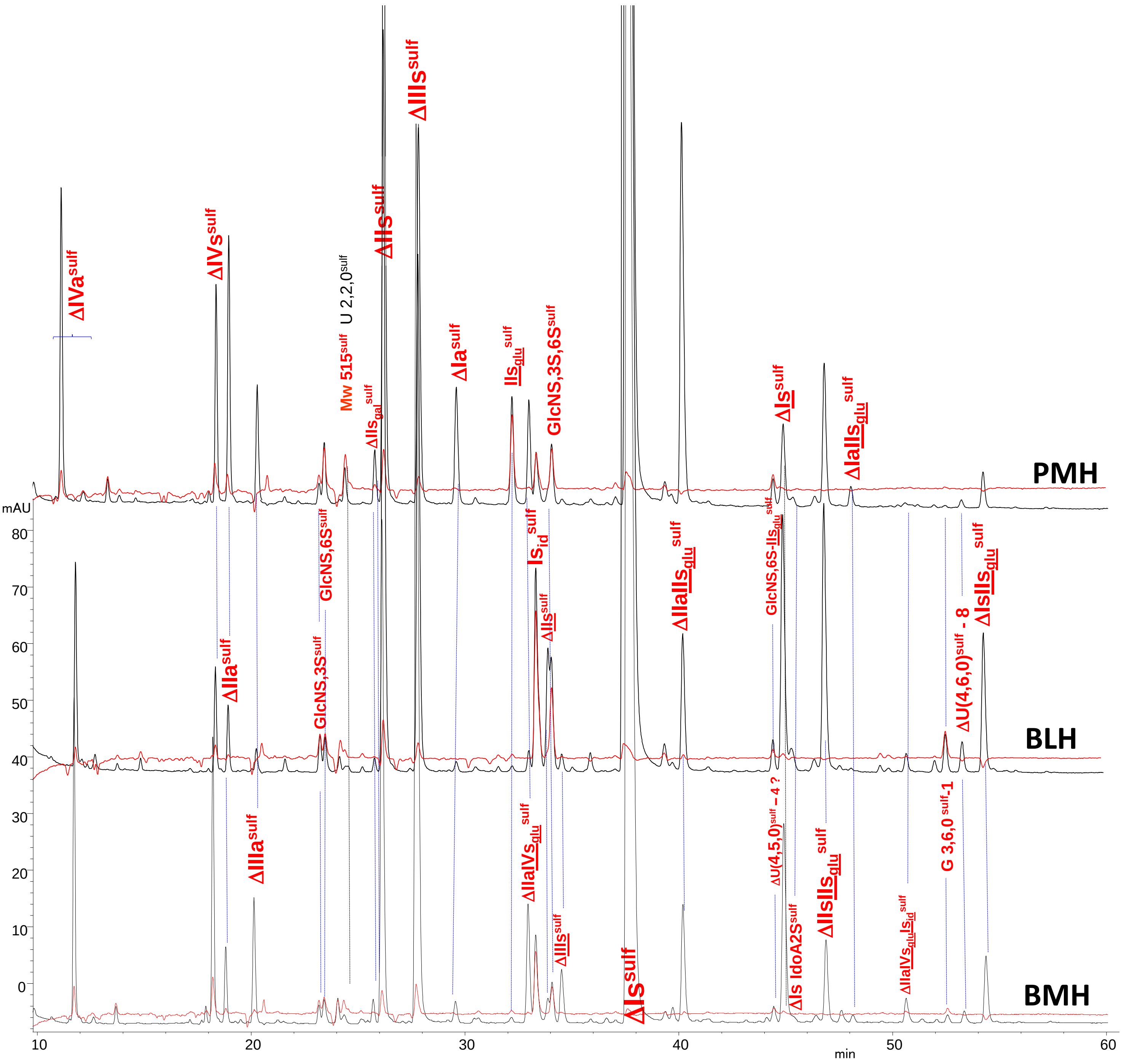

S-Figure 4: Chromatograms on AS11 of exhaustive heparinase digests of the ATIII high affinity fractions 5 (HA5) from 3 heparin sources (PMH, BLH, BMH) with sulfanilic tagging. Detection: — 265 nm; — 265 nm - 2.21 x 232 nm (UV selective saturated NRE signal).

1.2 Digests by heparinase II

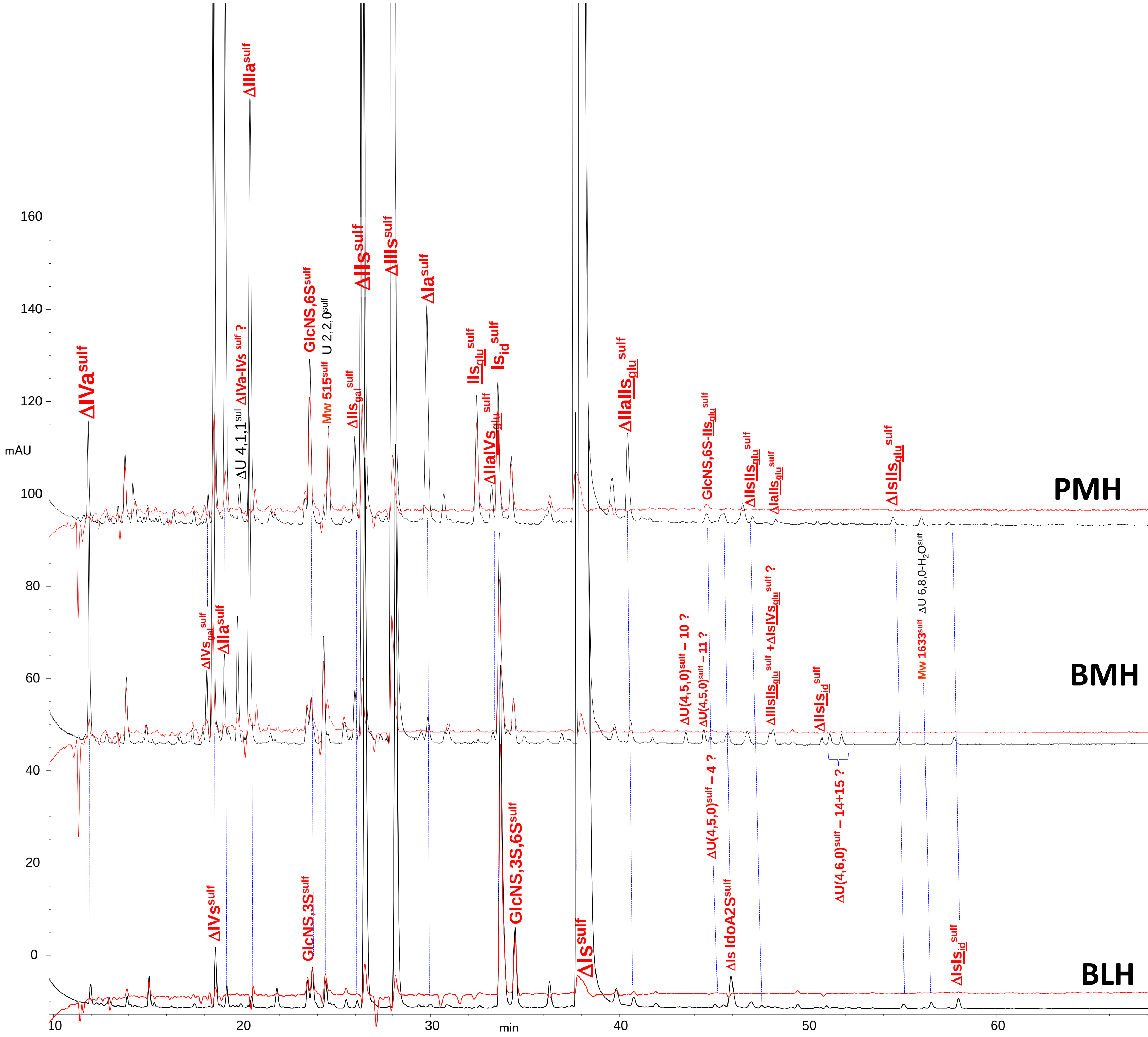

S-Figure 5: Chromatograms on AS11 of heparinase II digests of the ATIII low affinity (LA) fractions from 3 heparin sources (BLH, BMH, PMH) with sulfanilic tagging. Detection: — 265 nm; — 265 nm - 2.21 x 232 nm (UV selective saturated NRE signal).

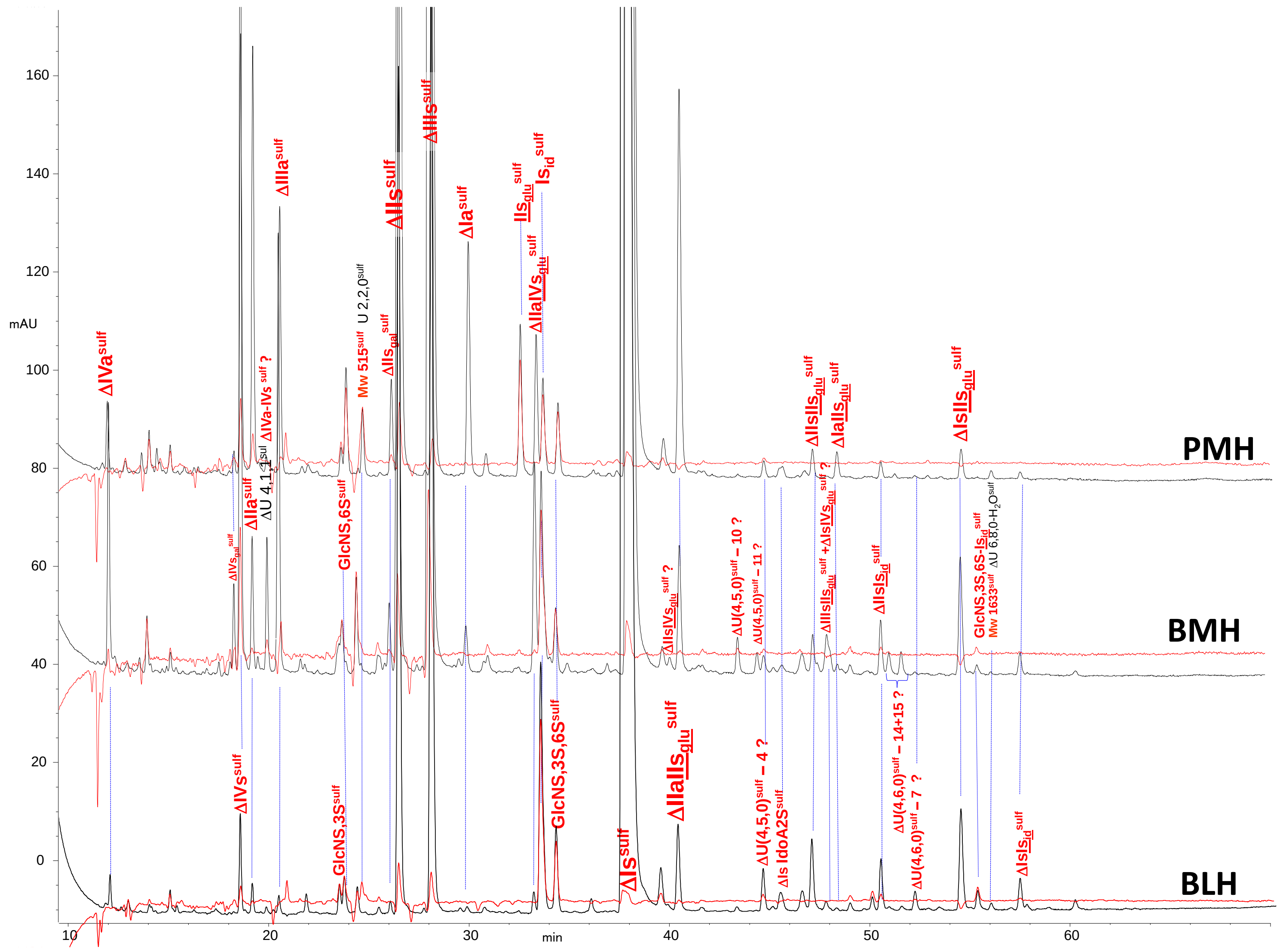

S-Figure 6: Chromatograms on AS11 of heparinase II digests of the ATIII high affinity fractions 1 (HA1) from 3 heparin sources (BLH, BMH, PMH) with sulfanilic tagging. Detection: — 265 nm; — 265 nm - 2.21 x 232 nm (UV selective saturated NRE signal).

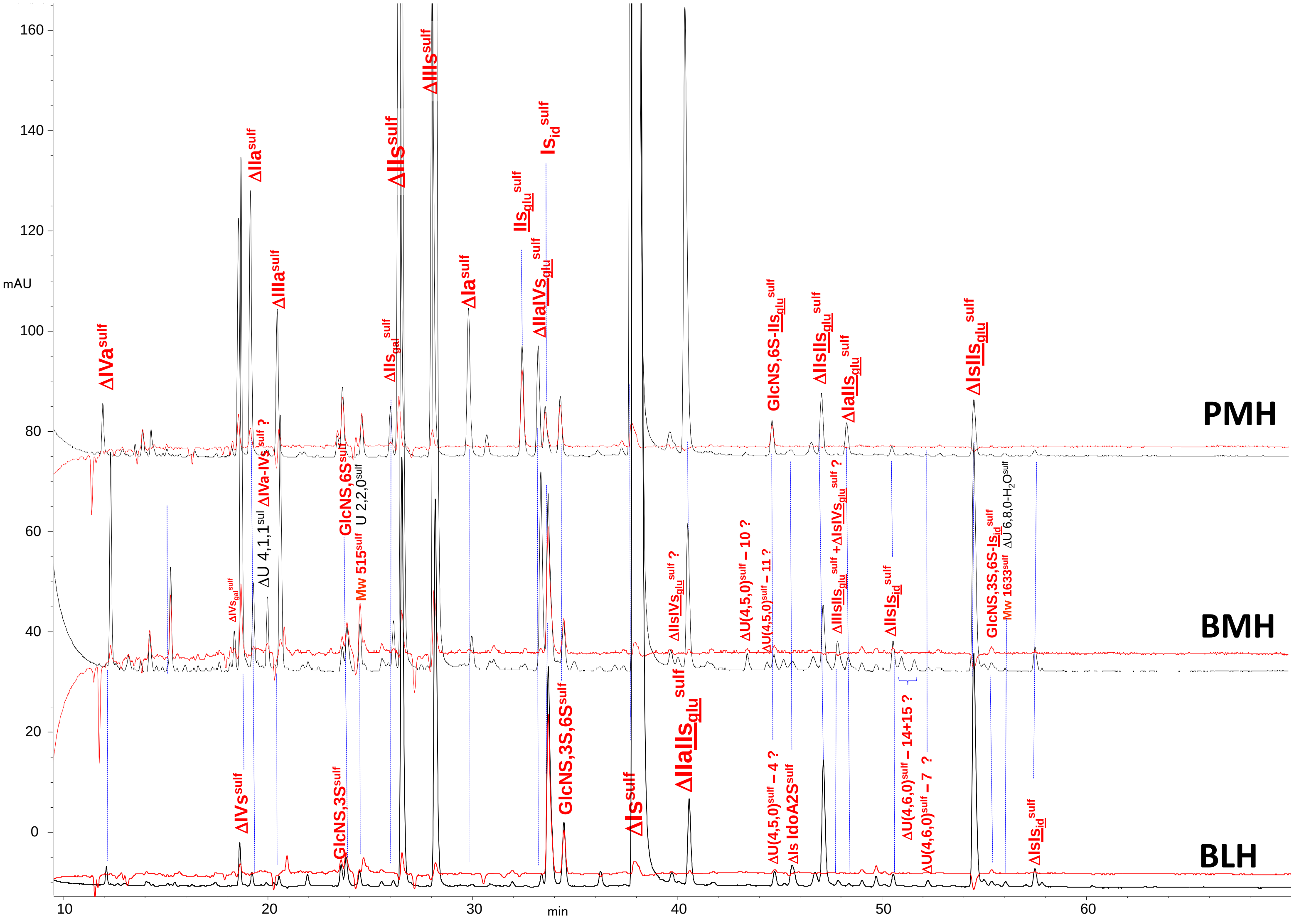

S-Figure 7: Chromatograms on AS11 of heparinase II digests of the ATIII high affinity fractions 3 (HA3) from 3 heparin sources (BLH, BMH, PMH) with sulfanilic tagging. Detection: — 265 nm; — 265 nm - 2.21 x 232 nm (UV selective saturated NRE signal).

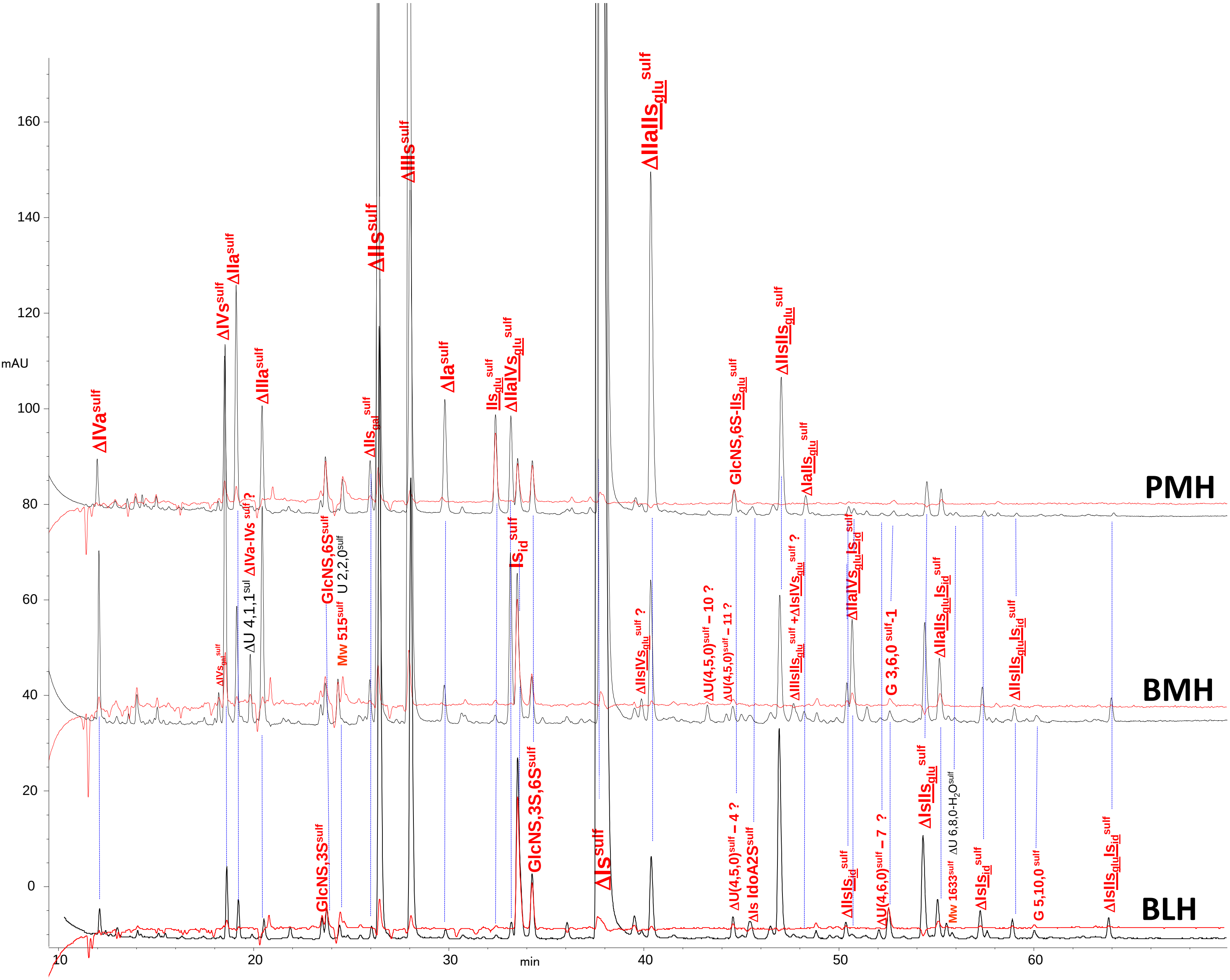

S-Figure 8: Chromatograms on AS11 of heparinase II digests of the ATIII high affinity fractions 5 (HA5) from 3 heparin sources (BLH, BMH, PMH) with sulfanilic tagging. Detection: — 265 nm; — 265 nm - 2.21 x 232 nm (UV selective saturated NRE signal).

2 Influence of the digestion (heparinase I+II+III/heparinase II only)

2.1 Porcine mucosa heparin

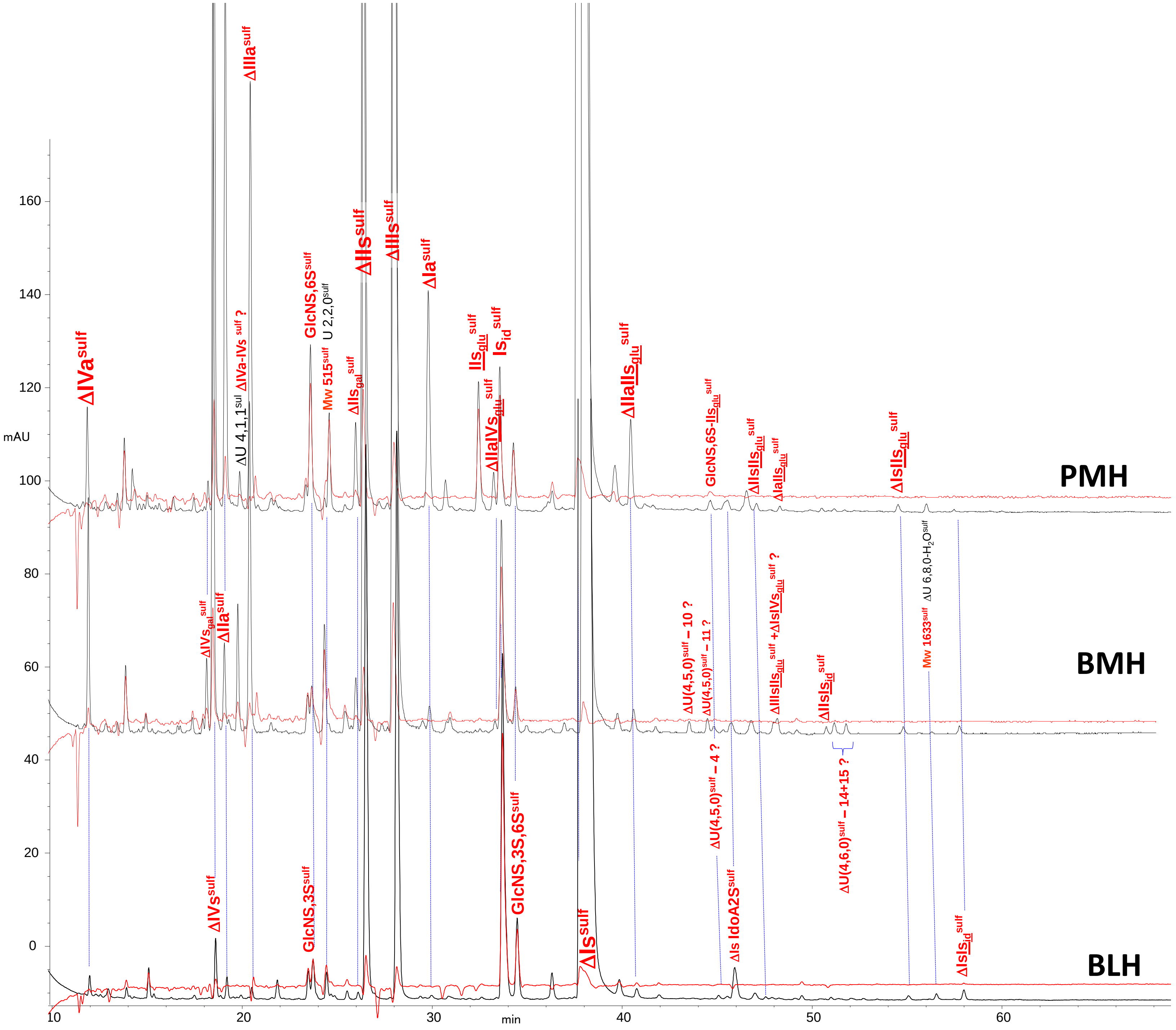

S-Figure 9: Comparison on AS11 method of heparinase I+II+III and heparinase II digests of porcine mucosal heparin (PMH) low-affinity (LA) fraction after sulfanilic reductive amination. Detection: — 265 nm; — 265 nm - 2.21 x 232 nm (UV selective saturated NRE signal).

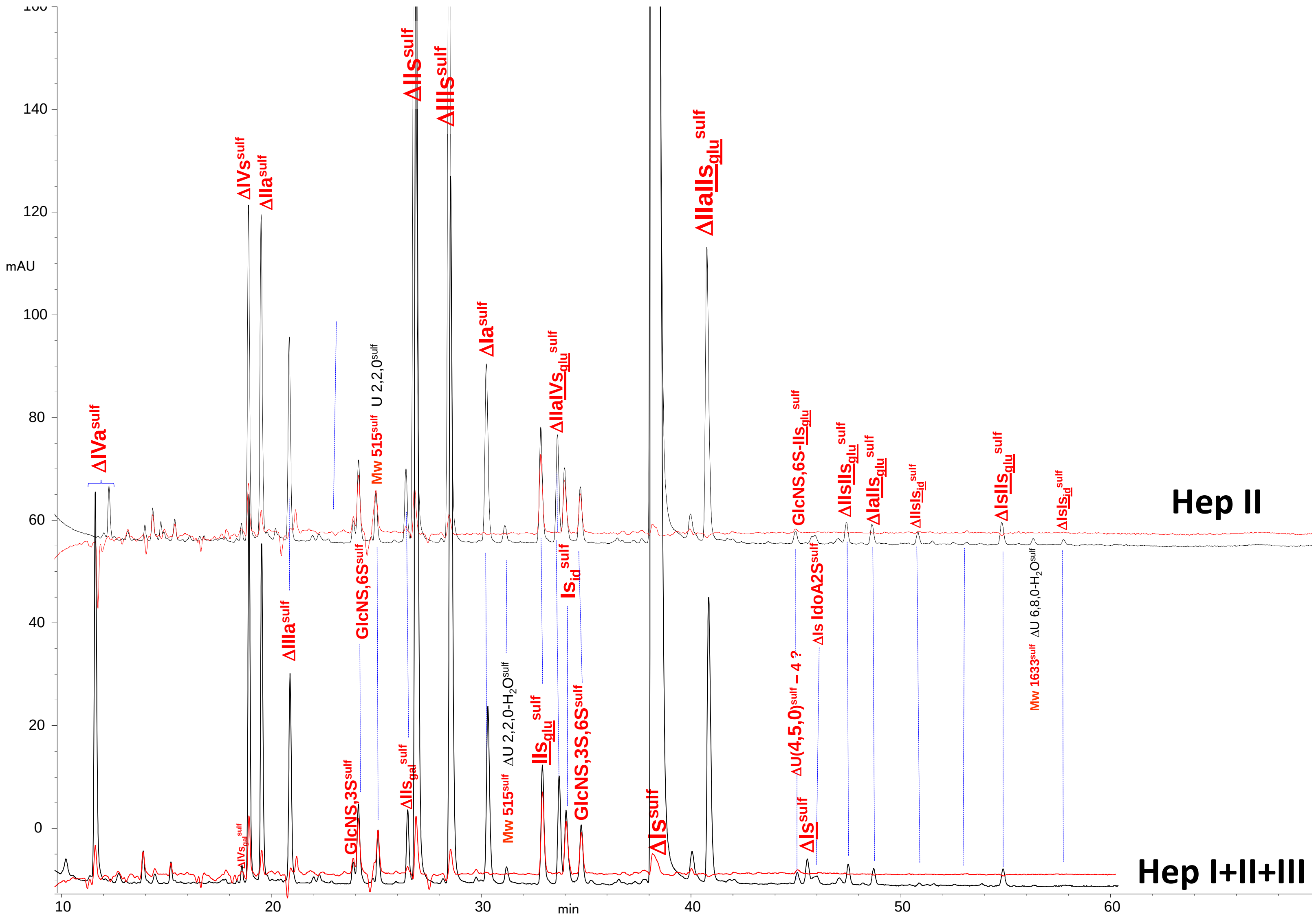

S-Figure 10: Comparison on AS11 method of heparinase I+II+III and heparinase II digests of porcine mucosal heparin (PMH) high-affinity (HA1) fraction after sulfanilic reductive amination. Detection: — 265 nm; — 265 nm - 2.21 x 232 nm (UV selective saturated NRE signal).

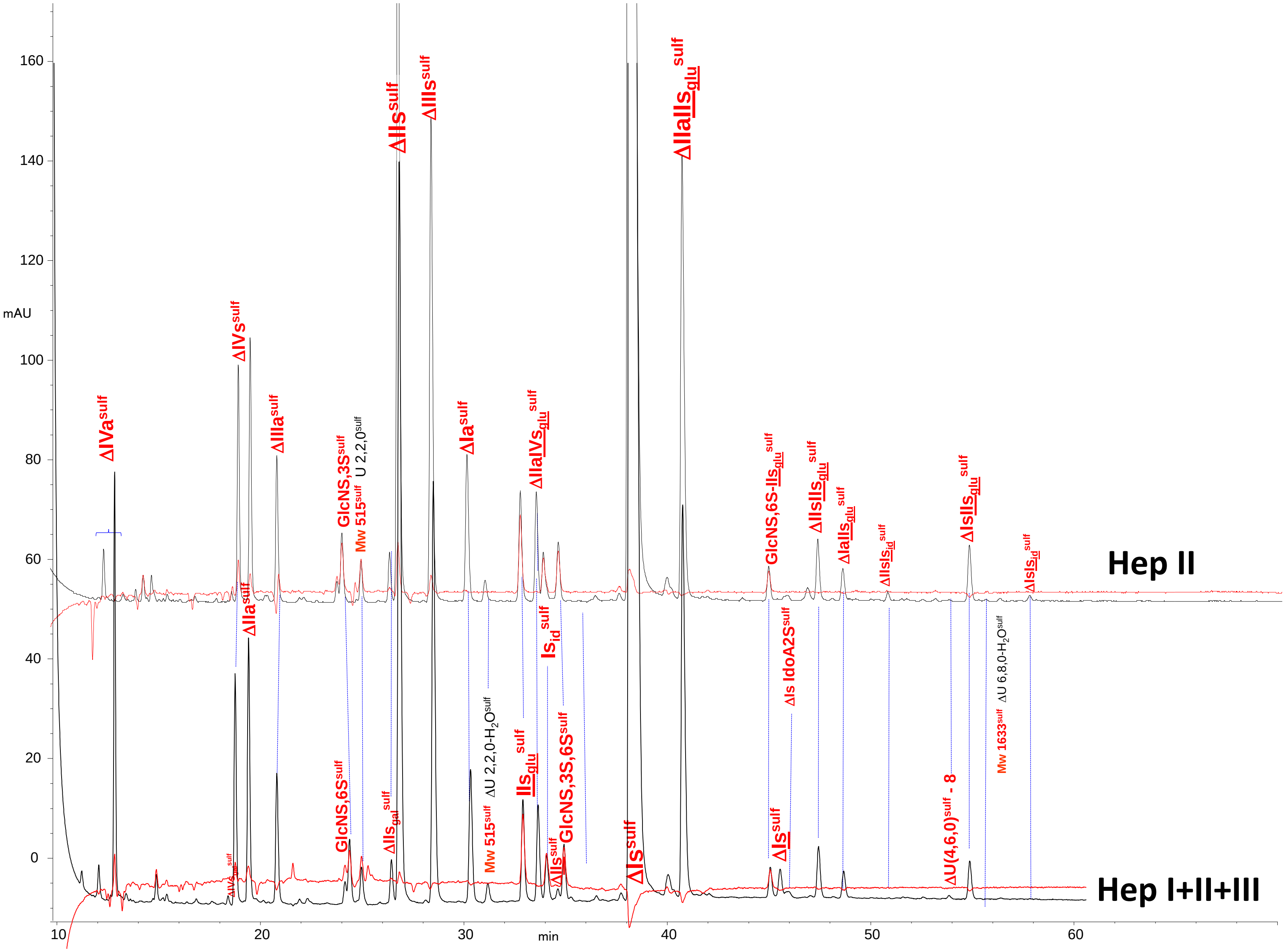

S-Figure 11: Comparison on AS11 method of heparinase I+II+III and heparinase II digests of porcine mucosal heparin (PMH) high-affinity (HA3) fraction after sulfanilic reductive amination. Detection: — 265 nm; — 265 nm - 2.21 x 232 nm (UV selective saturated NRE signal).

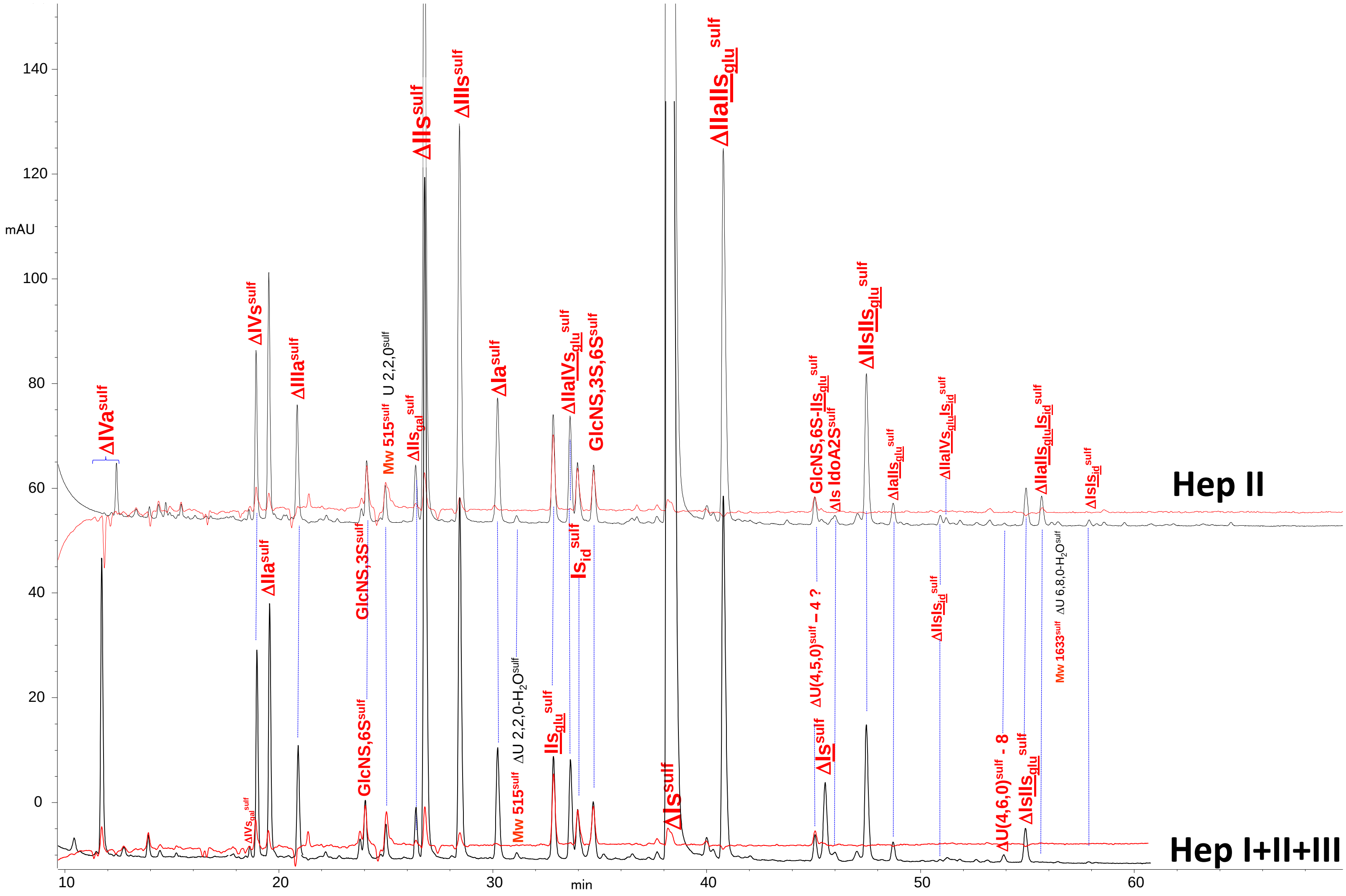

S-Figure 12: Comparison on AS11 method of heparinase I+II+III and heparinase II digests of porcine mucosal heparin (PMH) high-affinity (HA5) fraction after sulfanilic reductive amination. Detection: — 265 nm; — 265 nm - 2.21 x 232 nm (UV selective saturated NRE signal).

2.2 Bovine Mucosa Heparin

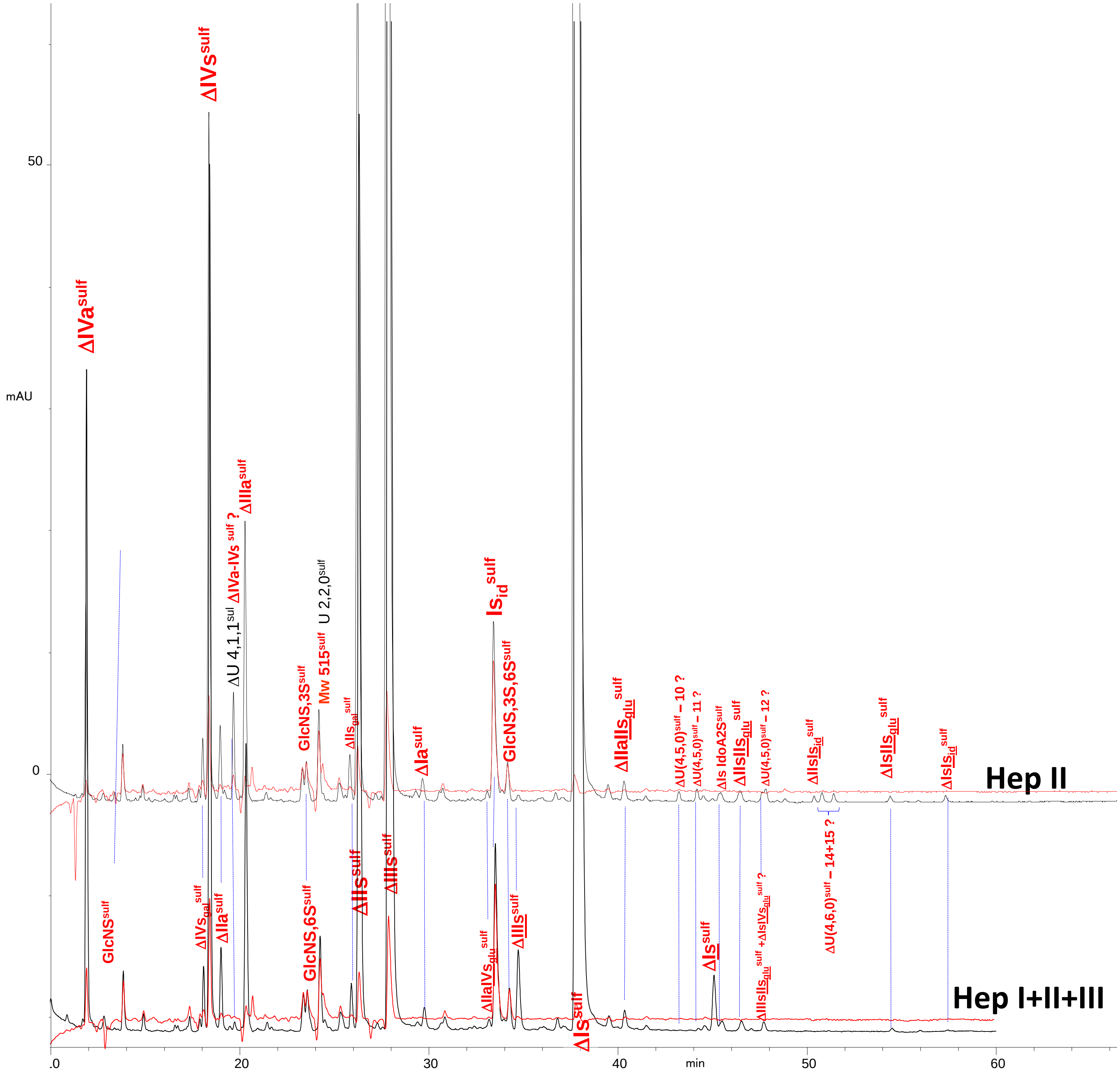

S-Figure 13: Comparison on AS11 method of heparinase I+II+III and heparinase II digests of bovine mucosal heparin (BMH) low-affinity (LA) fraction after sulfanilic reductive amination. Detection: — 265 nm; — 265 nm - 2.21 x 232 nm (UV selective saturated NRE signal).

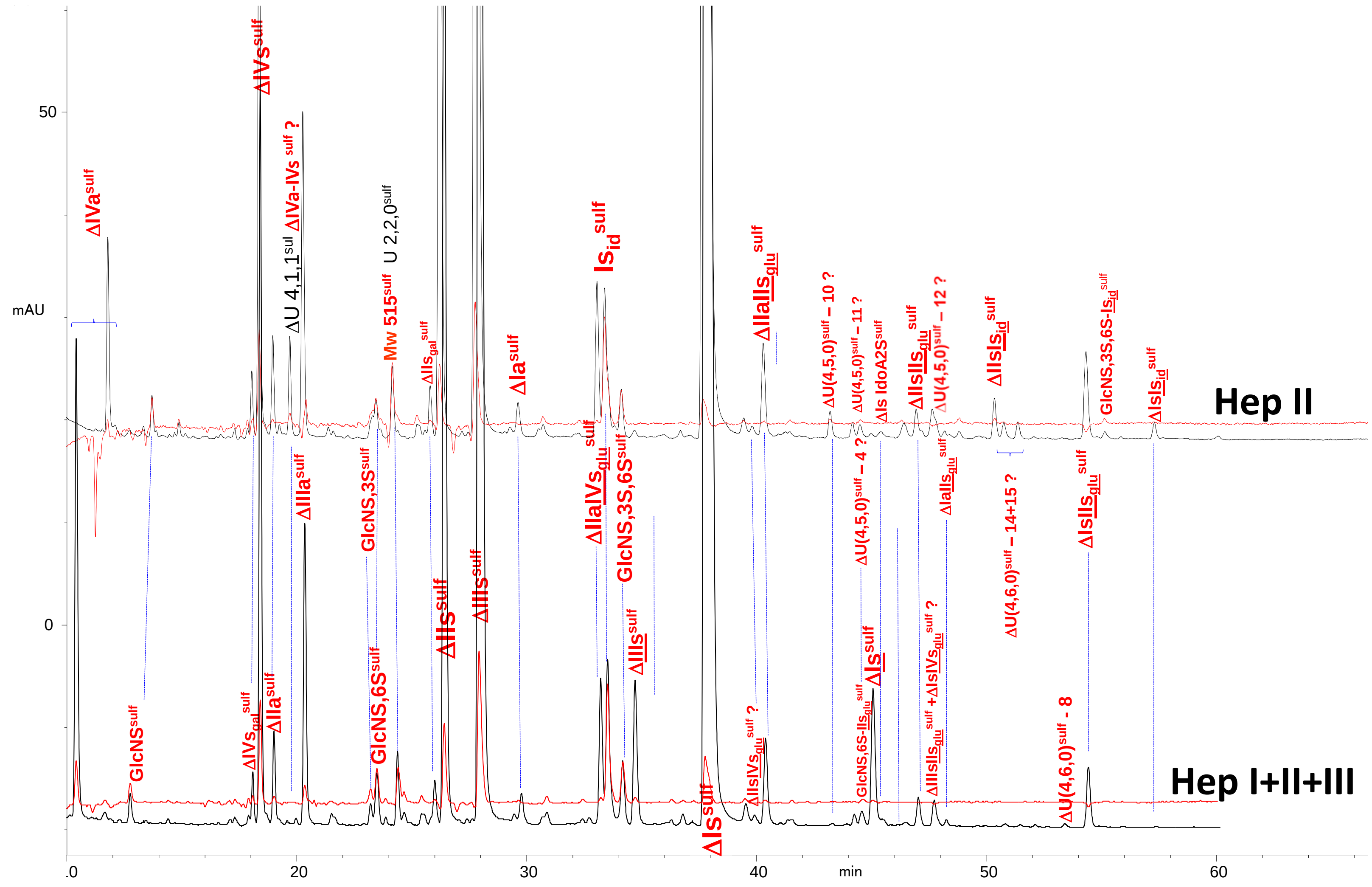

S-Figure 14: Comparison on AS11 method of heparinase I+II+III and heparinase II digests of bovine mucosal heparin (BMH) high-affinity (HA1) fraction after sulfanilic reductive amination. Detection: — 265 nm; — 265 nm - 2.21 x 232 nm (UV selective saturated NRE signal).

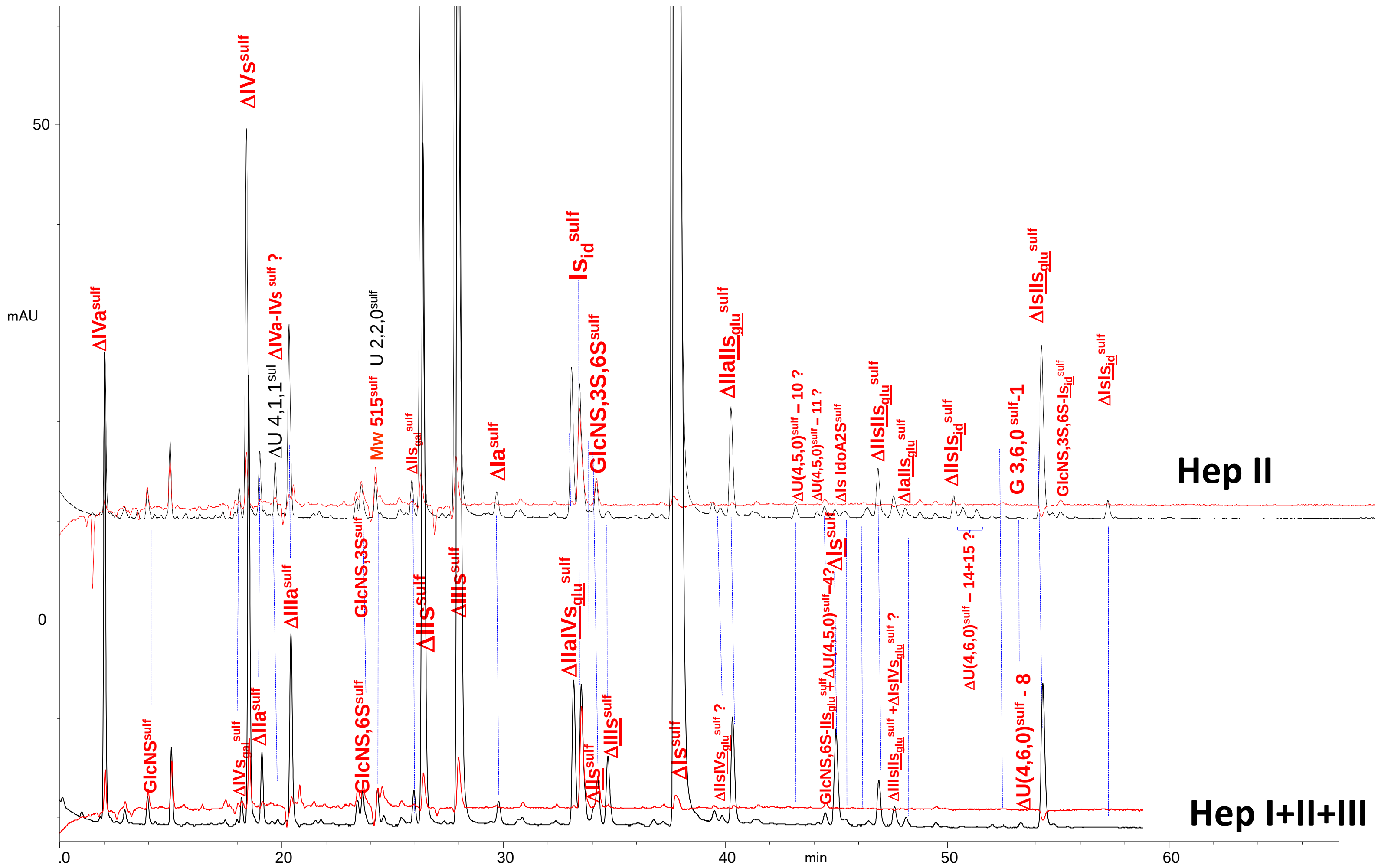

S-Figure 15: Comparison on AS11 method of heparinase I+II+III and heparinase II digests of bovine mucosal heparin (BMH) high-affinity (HA3) fraction after sulfanilic reductive amination. Detection: — 265 nm; — 265 nm - 2.21 x 232 nm (UV selective saturated NRE signal).

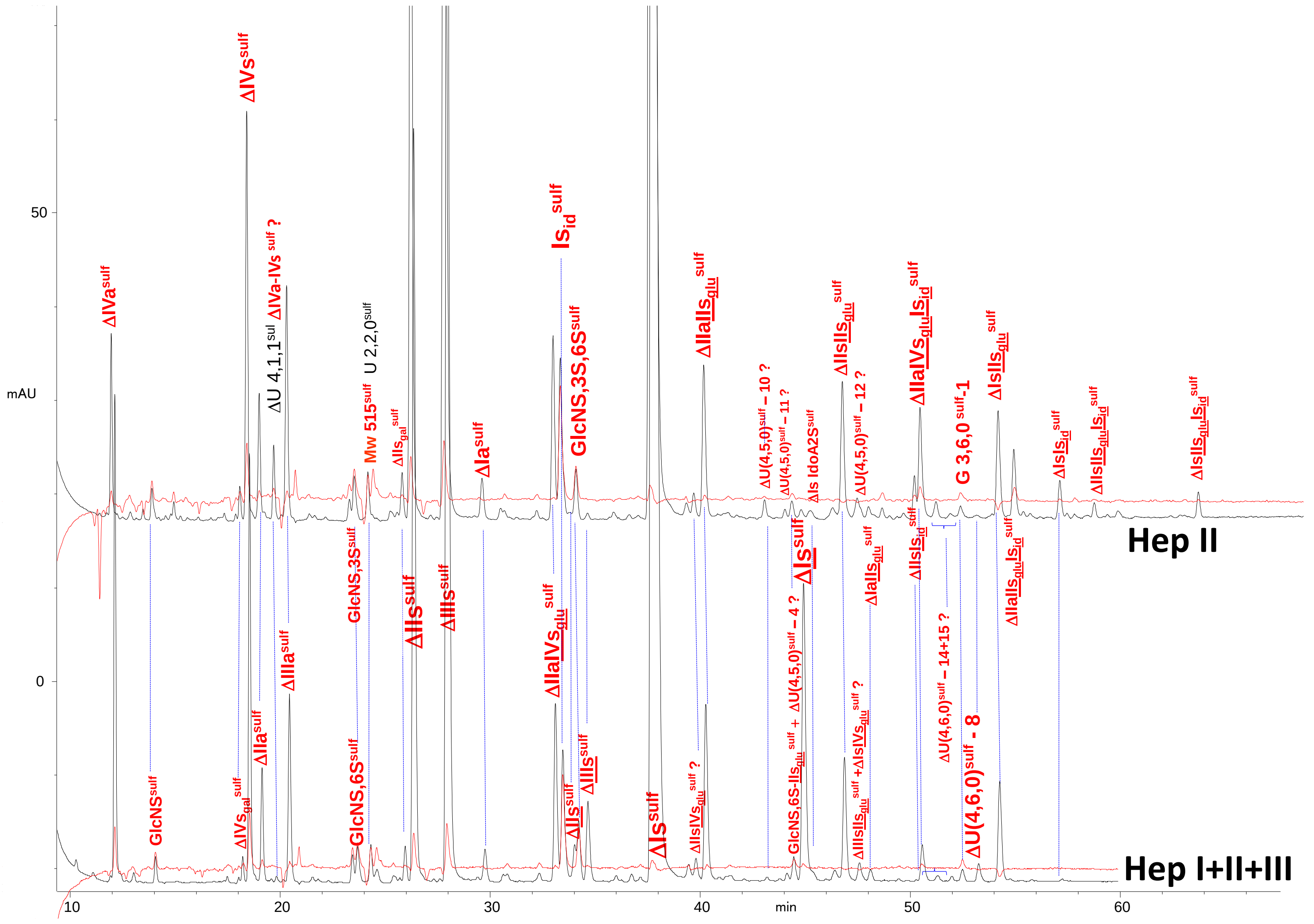

S-Figure 16: Comparison on AS11 method of heparinase I+II+III and heparinase II digests of bovine mucosal heparin (BMH) high-affinity (HA5) fraction after sulfanilic reductive amination. Detection: — 265 nm; — 265 nm - 2.21 x 232 nm (UV selective saturated NRE signal).

2.3 Bovine Lung heparin

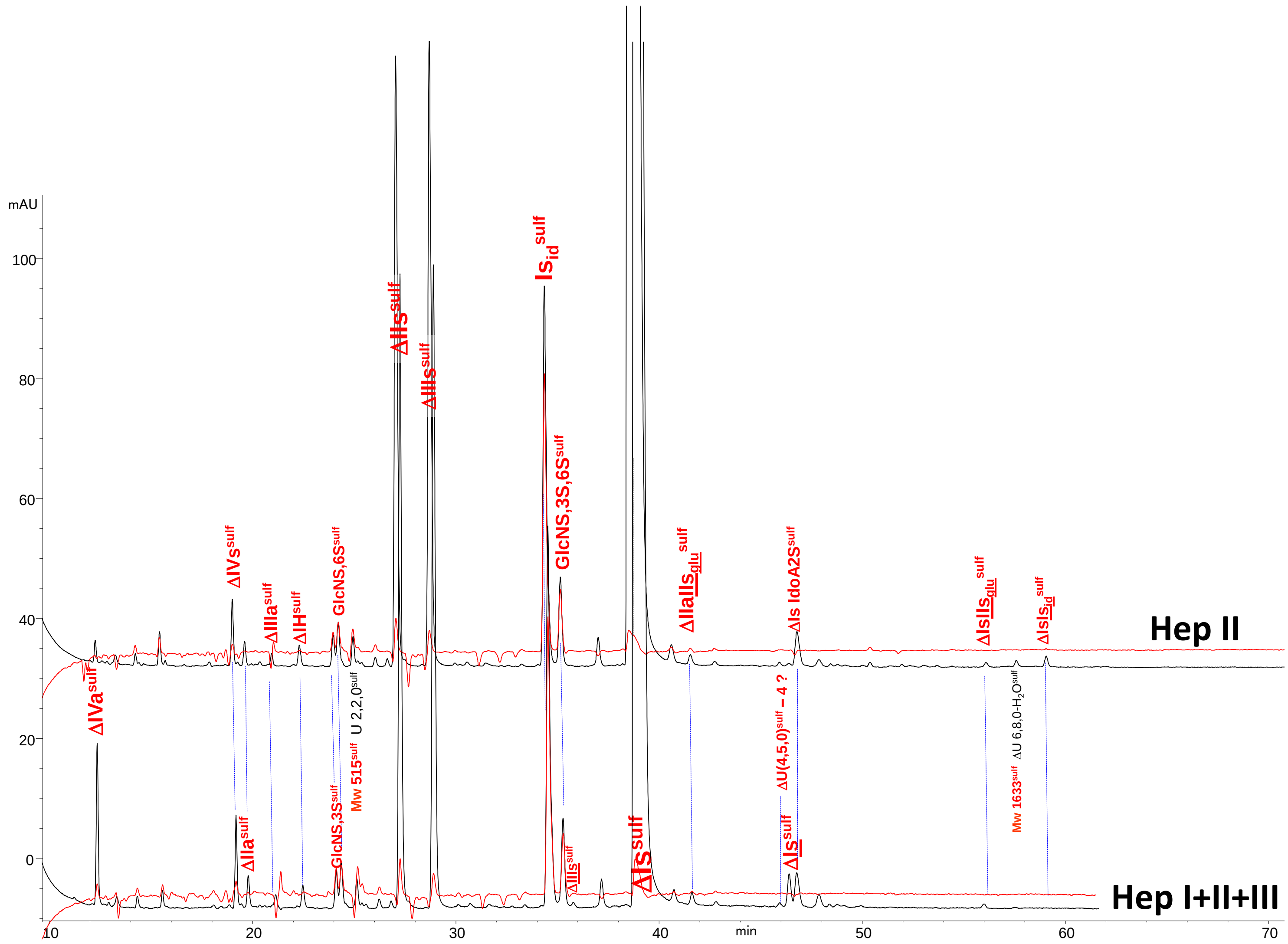

S-Figure 17: Comparison on AS11 method of heparinase I+II+III and heparinase II digests of bovine lung heparin (BLH) low-affinity (LA) fraction after sulfanilic reductive amination. Detection: — 265 nm; — 265 nm - 2.21 x 232 nm (UV selective saturated NRE signal).

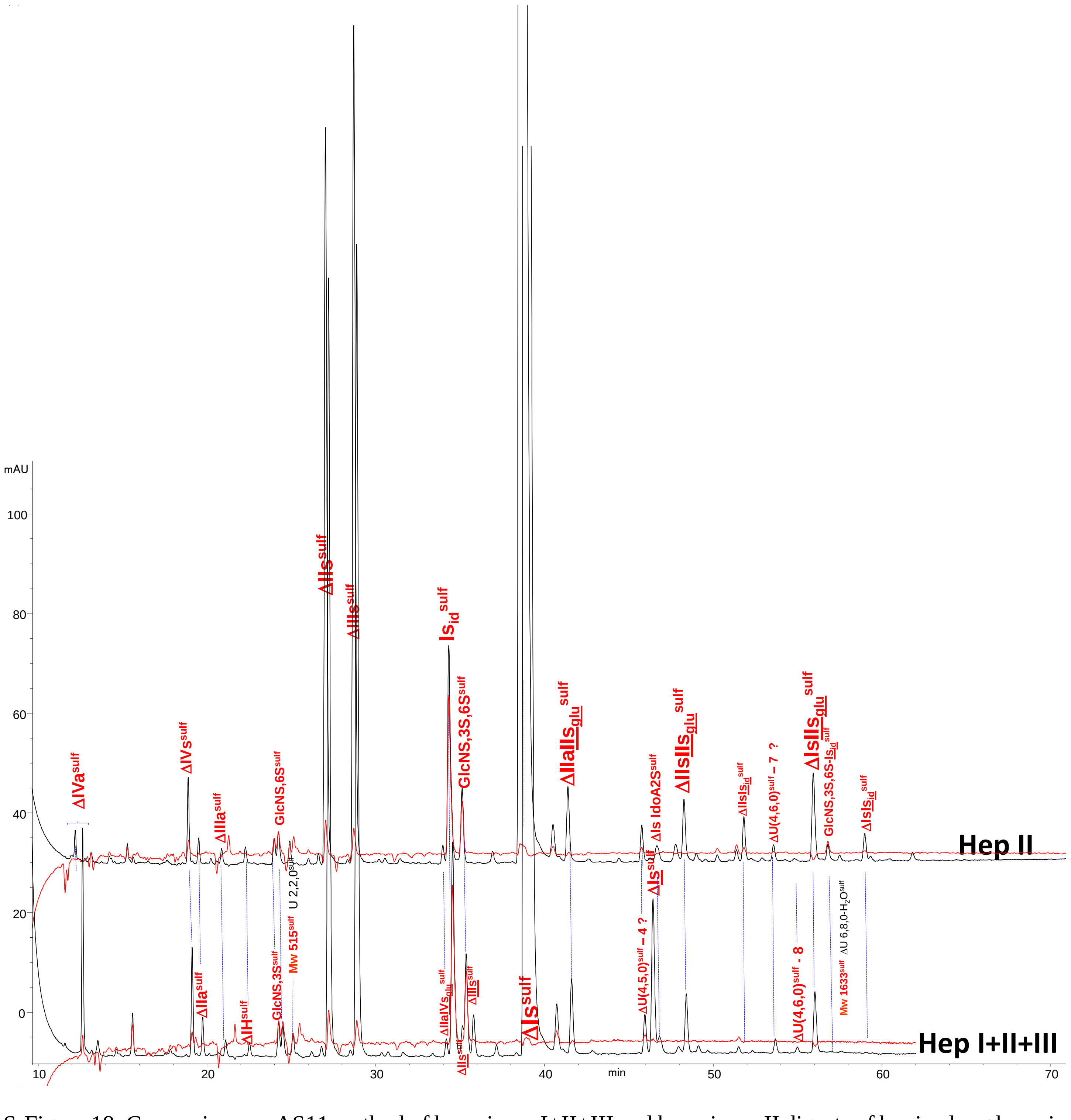

S-Figure 18: Comparison on AS11 method of heparinase I+II+III and heparinase II digests of bovine lung heparin (BLH) high-affinity (HA1) fraction after sulfanilic reductive amination. Detection: — 265 nm; — 265 nm - 2.21 x 232 nm (UV selective saturated NRE signal).

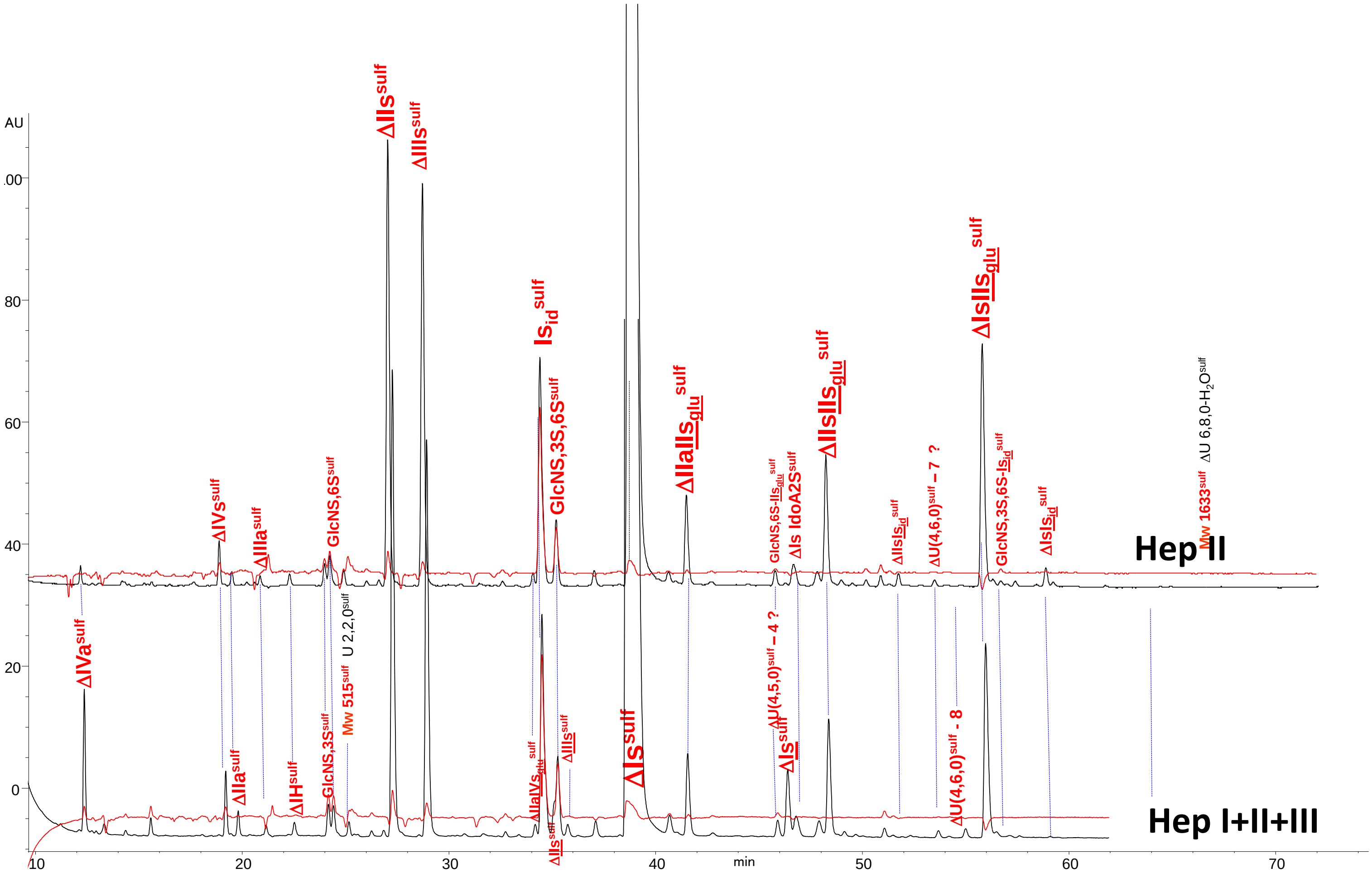

S-Figure 19: Comparison on AS11 method of heparinase I+II+III and heparinase II digests of bovine lung heparin (BLH) high-affinity (HA3) fraction after sulfanilic reductive amination. Detection: — 265 nm; — 265 nm - 2.21 x 232 nm (UV selective saturated NRE signal).

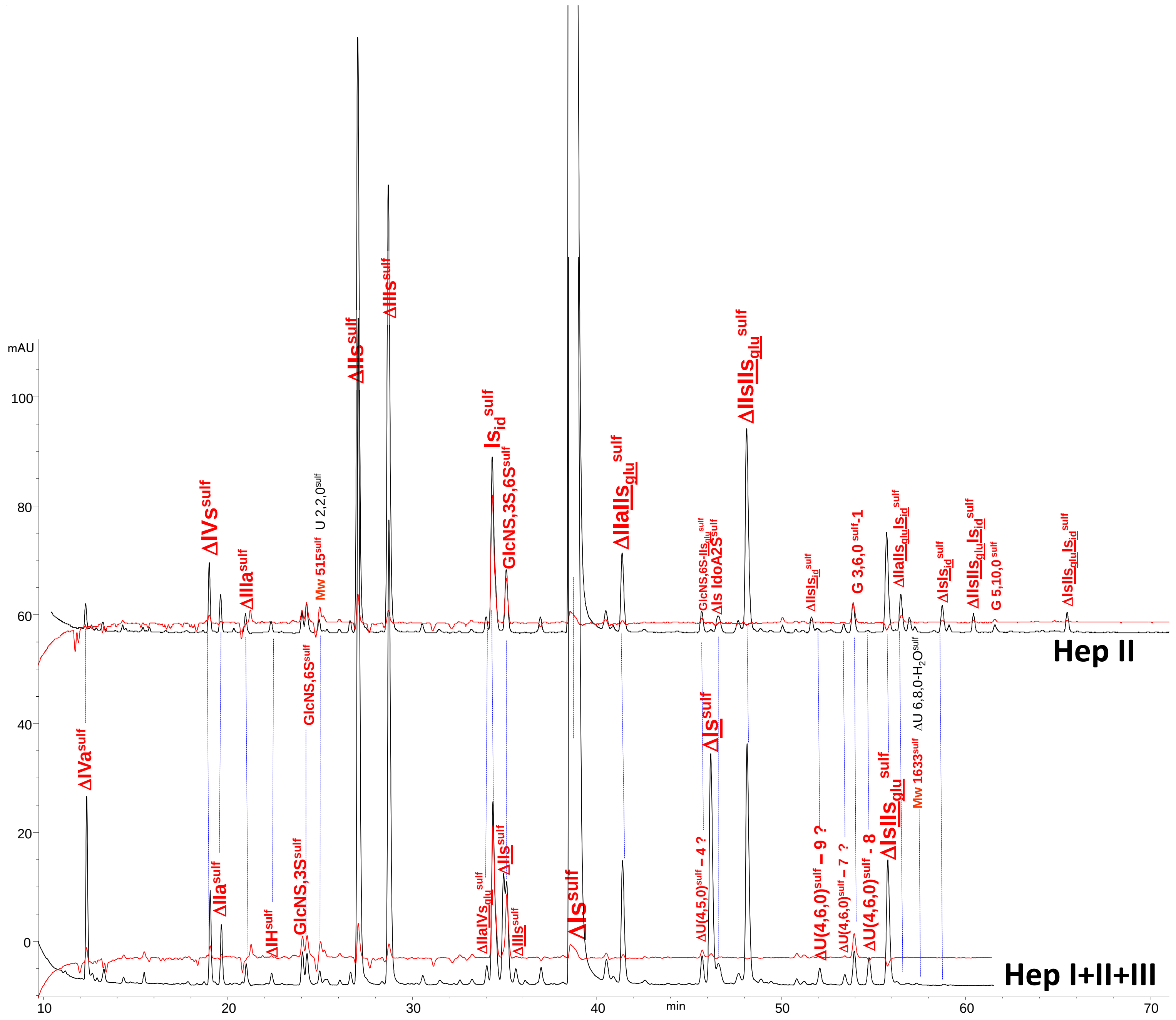

S-Figure 20: Comparison on AS11 method of heparinase I+II+III and heparinase II digests of bovine lung heparin (BLH) high-affinity (HA5) fraction after sulfanilic reductive amination. Detection: — 265 nm; — 265 nm - 2.21 x 232 nm (UV selective saturated NRE signal).
